# Frequent Drivers, Occasional Passengers: Signals of Symbiont-Driven Seasonal Adaptation and Hitchhiking in the Pea Aphid, *Acyrthosiphon pisum*

**DOI:** 10.3390/insects12090805

**Published:** 2021-09-08

**Authors:** Melissa Carpenter, Linyao Peng, Andrew H. Smith, Jonah Joffe, Michael O’Connor, Kerry M. Oliver, Jacob A. Russell

**Affiliations:** 1 Department of Biodiversity, Earth, and Environmental Science, Drexel University, 3250 Chestnut St., Philadelphia, PA 19104, USA; mmc435@drexel.edu (M.C.); andrew.smith@rodaleinstitute.org (A.H.S.); oconnomp@drexel.edu (M.O.); 2 Department of Biology, Drexel University, 3245 Chestnut St., Philadelphia, PA 19104, USA; lp595@drexel.edu (L.P.); jonah.joffe@gmail.com (J.J.); 3 Department of Entomology, University of Georgia, 120 Cedar St., Athens, GA 30602, USA; kmoliver@uga.edu

**Keywords:** adaptation, symbiont, bacteria, *Wolbachia*, aphid, hitchhiking

## Abstract

**Simple Summary:**

Rapid adaptation has been observed for several insects, including a number of agricultural pests. In these instances, it is expected that variation in insect-encoded genes encodes the variable phenotypes being acted on by natural selection. However, in addition to the thousands of genes encoded in their genomes, many insects harbor maternally transmitted, symbiotic bacteria encoding hundreds to thousands of genes of their own. Variation in the genes or presence and absence of such bacteria may, thus, also cause phenotypic variation, providing further raw material for natural selection. Here we studied symbiotic bacteria of the pea aphid, demonstrating that several change in prevalence within a single growing season. The frequencies of some bacteria shifted in concert with environmental factors predicted to determine their costs and benefits. Interpreting these correlations as plausible signals of symbiont-mediated insect adaptation, we found little evidence in support of alternative hypotheses, and that defense against fungal pathogens was possibly the most common beneficial symbiont service. Yet we also found that the particular combinations of bacteria living within an aphid may sometimes shape how aphids respond to natural selection. Our results have implications for the management of crop pests and for understanding the nature of rapid insect adaptation.

**Abstract:**

Insects harbor a variety of maternally inherited bacterial symbionts. As such, variation in symbiont presence/absence, in the combinations of harbored symbionts, and in the genotypes of harbored symbiont species provide heritable genetic variation of potential use in the insects’ adaptive repertoires. Understanding the natural importance of symbionts is challenging but studying their dynamics over time can help to elucidate the potential for such symbiont-driven insect adaptation. Toward this end, we studied the seasonal dynamics of six maternally transferred bacterial symbiont species in the multivoltine pea aphid (*Acyrthosiphon pisum*). Our sampling focused on six alfalfa fields in southeastern Pennsylvania, and spanned 14 timepoints within the 2012 growing season, in addition to two overwintering periods. To test and generate hypotheses on the natural relevance of these non-essential symbionts, we examined whether symbiont dynamics correlated with any of ten measured environmental variables from the 2012 growing season, including some of known importance in the lab. We found that five symbionts changed prevalence across one or both overwintering periods, and that the same five species underwent such frequency shifts across the 2012 growing season. Intriguingly, the frequencies of these dynamic symbionts showed robust correlations with a subset of our measured environmental variables. Several of these trends supported the natural relevance of lab-discovered symbiont roles, including anti-pathogen defense. For a seventh symbiont—*Hamiltonella defensa*—studied previously across the same study periods, we tested whether a reported correlation between prevalence and temperature stemmed not from thermally varying host-level fitness effects, but from selection on co-infecting symbionts or on aphid-encoded alleles associated with this bacterium. In general, such “hitchhiking” effects were not evident during times with strongly correlated *Hamiltonella* and temperature shifts. However, we did identify at least one time period in which *Hamiltonella* spread was likely driven by selection on a co-infecting symbiont—*Rickettsiella viridis*. Recognizing the broader potential for such hitchhiking, we explored selection on co-infecting symbionts as a possible driver behind the dynamics of the remaining six species. Out of twelve examined instances of symbiont dynamics unfolding across 2-week periods or overwintering spans, we found eight in which the focal symbiont underwent parallel frequency shifts under single infection and one or more co-infection contexts. This supported the idea that phenotypic variation created by the presence/absence of individual symbionts is a direct target for selection, and that symbiont effects can be robust under co-habitation with other symbionts. Contrastingly, in two cases, we found that selection may target phenotypes emerging from symbiont co-infections, with specific species combinations driving overall trends for the focal dynamic symbionts, without correlated change under single infection. Finally, in three cases—including the one described above for *Hamiltonella*—our data suggested that incidental co-infection with a (dis)favored symbiont could lead to large frequency shifts for “passenger” symbionts, conferring no apparent cost or benefit. Such hitchhiking has rarely been studied in heritable symbiont systems. We propose that it is more common than appreciated, given the widespread nature of maternally inherited bacteria, and the frequency of multi-species symbiotic communities across insects.

## 1. Introduction

Populations of multivoltine organisms, living through more than one generation per year, can show remarkable genetic and phenotypic change within a single season [1,2]. This can stem from adaptive processes in which the benefits of alternative phenotypes, controlled by variable genomic loci, fluctuate with seasonally varying food quality, temperature, humidity, parasites, pathogens, or predators, e.g., [3,4]. While the driving environmental pressures and genetic bases are understood in some cases [5,6]. few systems have been studied in nature with the precision required to document such seasonal adaptation, a phenomenon that blurs the distinctions between ecological and evolutionary timescales.

Insects number among a diverse array of multivoltine animals. Many are also hosts to maternally inherited bacterial symbionts [7,8], an additional source of heritable variation with potential implications for seasonal adaptation. Heritable symbionts can have profound effects on insect phenotypes [9,10,11,12,13,14], shaping dietary utilization [15,16,17], and tolerance to a range of biotic and abiotic ecological stressors [18,19,20,21,22]. While field-based studies have shown their impacts on insect adaptation across multi-year durations [23,24], it is also possible that heritable symbionts shape more rapid, within-season adaptation in multivoltine insects [25]. 

As the insects’ best-studied and most widespread heritable symbiont [26,27,28,29,30], *Wolbachia* is a likely candidate for such adaptive impacts. But *Wolbachia* is not common in all insect species, suggesting the potential influence of several other taxa [31]. These include common, generalist symbionts from widely distributed bacterial genera like *Spiroplasma*, *Rickettsia*, *Cardinium*, *Sodalis*, and *Arsenophonus* [32,33,34,35,36]. Others come from more specialized lineages, as is the case for a subset of the heritable bacteria found in aphids (Hemiptera: Aphididae) and in their relatives within the Sternorrhyncha [37,38].

Apart from the obligate nutritional symbionts found in insects with imbalanced diets [39], most heritable symbionts are thought to be non-essential, or facultative, from the host insect’s perspective—a finding occasionally borne out through lab-based study [40,41,42,43]. Under certain circumstances, some facultative symbionts may evolve toward obligate associations and ubiquity [44,45,46,47,48]. But more typically, heritable facultative symbionts are found at intermediate prevalence within host populations [49,50,51]. Frequencies can approach fixation and stable equilibria for some such symbionts—including a subset of those employing reproductive manipulation [52]. In other cases, rather modest percentages of an insect population are infected by a given symbiont species. Frequencies of such bacteria can, further, be dynamic, implicating variable rates of maternal transmission or fitness effects that vary over time [53,54]. 

Dynamics of facultative symbionts can unfold across multi-year time scales [24,55,56,57], or—for some multivoltine insects—within a single season [58,59,60]. Using rates of frequency change over time, and knowledge of symbionts’ ecological roles, prior researchers have inferred selection (s) coefficients associated with symbiont infection [23,61,62]. But few studies have tried to relate within-season symbiont dynamics with environmentally imposed selective agents. In past research, we have attempted to fill this gap for the seven heritable bacterial symbionts found in United States populations of the pea aphid [60,63,64]. Through day-degree calculations, and prior temperature-calibrated studies of development time [65,66], we recently estimated a span of 16 generations for multivoltine pea aphids (*Acyrthosiphon pisum*) collected across a 26-week span in the 2012 Pennsylvania growing season [60]. The heritable bacterium *Hamiltonella defensa* underwent broad shifts in prevalence across this span. Remarkably, spikes and declines in *Hamiltonella* frequencies, occasionally exceeding 20% in magnitude, unfolded across 2-week intervals—a span of only 1–2 generations. After accounting for a range of plausible maternal transmission rates [67] and operating under the assumption that symbiont frequency shifts reflect direct selection on phenotypic differences created by *Hamiltonella* presence/absence, we determined that selection (s) coefficients for *Hamiltonella*-infected aphids changed sign within the 2012 season, supporting a role for seasonal balancing selection in sustaining this symbiont’s infection polymorphism within some aphid populations [60]. 

We originally hypothesized that within-season dynamics for this symbiont would be driven by seasonally varying selective pressures imposed by parasitoids [25,64]. Indeed, in the laboratory, nearly all studied strains of *Hamiltonella* confer some level of physiological defense to pea aphids after parasitism by the braconid parasitoid wasp, *Aphidius ervi* [68]. Accordingly, field studies have identified wasp parasitism as a likely driver *of H. defensa* infection frequencies [64,69]. But in our prior 2012 investigation, neither the prevalence of *A. ervi*, nor its rates of successful pea aphid parasitism, were predictive of *Hamiltonella* prevalence [60]. 

Instead, across this season, and in two subsequent overwintering periods, *Hamiltonella* frequencies correlated positively with temperature. This finding was not consistent with the prediction that anti-parasitoid defense, varying pressures from parasitoids, and symbiont-imposed costs in their absence [70], are the primary drivers of *Hamiltonella* dynamics. A modest-at-best role for parasitoids was further bolstered by the knowledge that *Hamiltonella* protection fails at warmer temperatures [71,72], and that *Hamiltonella* was most common at the hottest times during the summer of 2012. Such *Hamiltonella* vs. temperature correlations were consistent with lab studies reporting that this symbiont may confer tolerance to the damaging effects of heat shock; that it provides benefits under rearing at constant, warm temperatures (e.g. 25 °C); or that it can be harmful under cooler conditions [73,74,75,76]. When combined with field cage studies verifying that parasitism can drive subtle impacts on *Hamiltonella* prevalence [60], our findings suggest multiple ecological roles for this symbiont under field conditions experienced in Pennsylvania.

Another factor, not accounted for in our prior study [60], was the potential for *Hamiltonella* to hitchhike alongside another component of pea aphid biology that was, instead, the target of selection. Often documented for linked loci in eukaryotic nuclear genomes, sweeps of heritable symbionts throughout insect populations have driven hitchhiking in mtDNA, due to their shared avenue of maternal transmission [77,78]. In the pea aphid, hitchhiking by symbionts like *Hamiltonella* could, conversely, unfold due to selection on some other linked maternally inherited element, whether this be mtDNA, varying strains of the obligate *Buchnera aphidicola* symbiont, or co-infections with any of the six remaining facultative symbionts [63]. Since pea aphids reproduce asexually during summer months, and since some clones may persist as asexual lineages across years [67], it is also possible that selection on particular aphid clones, incidentally infected with *Hamiltonella*, could enrich *Hamiltonella* frequencies via hitchhiking. In the present manuscript we examine these possibilities as part of a broader effort to study the forces shaping the maintenance of this ecologically impactful symbiont, e.g., [79,80,81].

In addition to *Hamiltonella*, the six other known heritable, facultative symbionts of pea aphids in Pennsylvania are *Regiella insecticola*, *Fukatsuia symbiotica*, *Rickettsiella viridis*, *Serratia symbiotica*, and two unnamed species from the genera *Spiroplasma* and *Rickettsia*. The former three are from lineages that appear largely restricted to aphids and their relatives [31,82], while the latter two hail from clades associated with a broad range of host taxa [34,83]. Phylogenetic analysis of *Serratia symbiotica* from pea aphids suggest that *these* symbionts are most closely related to other heritable facultative symbionts from varying aphids. This lineage is, in turn, related to *Serratia* clades with broader distributions across aphids, other insects, and the plant environment [84], and to (co-)obligate *Serratia* symbionts of Lachninae aphids [85].

Prior studies have suggested varying ecological roles for these additional pea aphid symbionts, including defense against the fungal pathogen *Pandora neoaphidis* (*Regiella*, *Rickettsia*, *Rickettsiella*, and some strains of *Spiroplasma—*e.g., [86,87]), thermotolerance (*Serratia* and *Rickettsia*, e.g., [74,88,89]), and possible further defense against *Aphidius ervi* or other parasitoids (*Spiroplasma* [90,91]). *Fukatsuia* symbionts have been shown to impact all three phenotypes in European pea aphids [92,93]. Beyond these services—which may boost aphid fitness under some field conditions [69]—costs of these symbionts have been identified [43]. These vary in their severity, and in the range of ecological conditions under which they have been seen, in both the lab [74,75,90,94,95] and the field [69,79,80]. As for *Hamiltonella*, the known seasonality of the factors predicted to shape these symbionts’ helpful vs. harmful nature leads to the prediction that they too may vary in prevalence across a single year, and that the diverse suite of harbored facultative symbionts may be part of an adaptive toolbox for the multivoltine pea aphid.

In the present study we assessed the ubiquity of seasonal dynamics for heritable facultative symbionts of pea aphids, the driving environmental forces behind such fluctuations, and the potential for symbiont hitchhiking, through a longitudinal field study performed in 2012. Following up on a smaller investigation of the same population in 2011 [64], we improved upon our original study through greater field replication, more frequent sampling, and measurement of a greater number of environmental variables. In addition, we expand on our recent 2012-focused, *Hamiltonella*-centric paper [60] through inclusion of the six remaining facultative symbionts. 

Having shown the aforementioned correlation between *Hamiltonella* and temperature, and hence the potential for rapid *Hamiltonella*-mediated aphid adaptation [60], we use the present study to test the alternative hypothesis that *Hamiltonella* dynamics extended from hitchhiking. We begin by asking whether their season-wide correlations with temperature; their rapid ≥20% frequency shifts unfolding across 2-week timespans; and their frequency declines across overwintering periods [60] could best be explained by selection on facultative symbionts found in common co-infection with *Hamiltonella*. For one warm period (Times 6–7, 2–18 July), in which *Hamiltonella* frequencies rose by 26%, we used microsatellite genotyping of pea aphids to assess the potential for hitchhiking with particular aphid clones, and Sanger sequencing of the *ibpA Buchnera* gene promoter to look for hitchhiking with symbiont-hospitable, thermally tolerant obligate symbiont strains [96,97]. Our discoveries provide insight into the rapid dynamics of heritable insect microbiomes, while further establishing the adaptive potential of symbionts that may earn their keep through a range of ecological services.

## 2. Methods

We conducted a longitudinal field study on the multivoltine pea aphid and its seven facultative symbionts, testing for change in symbiont frequencies across the 2012 field season. To test a priori hypotheses on the role of host-level natural selection as a driver of symbiont dynamics, we collected simultaneous data from several components of the environment, assessing their abilities to explain symbiont frequencies over time. An abbreviated overview of this approach, and our results, can be found in our Graphical Abstract, in addition to our utilization of overwintering studies, and hitchhiking assessments to further dissect symbiont dynamics and their causes. As most of the below methods are identical to those in Smith et al., 2021 [60], we present them here in a slightly abbreviated form.

### 2.1. 2012 Aphid Sampling

Aphids were collected from six organic alfalfa fields in Berks County, Pennsylvania in 2012 (Table S1). Field sampling occurred on a bi-weekly schedule from 25th April through 25th October, yielding 14 total collection events. We utilized beat sampling to remove aphids from plants, shaking the stems and leaves of alfalfa plants over a tray. Dislodged aphids were then transferred to plant-filled containers for lab transport (mortality assays), or to tubes containing 95% ethanol (molecular symbiont screening), through use of a fine bristle paint brush. To minimize the chances of resampling the same clone we spaced collecting sites by ~20 m, preserving no more than 1 pink and 1 green pea aphid for molecular symbiont screening, and keeping no more than 1 of each color alive for mortality assays, from each site. At each time point we collected an average of 35.8 aphids per field for survival/mortality assays, across an average of 5.1 fields. We performed diagnostic PCR screening for symbionts on an average of 22.9 aphids from each field and time. These symbiont-surveyed aphids represented an average of 5.5 fields from each time point.

### 2.2. Insect Counts and Climatic Measurements

To measure temperature and humidity—with the latter variable modeled here as vapor deficit (aridity)—we placed one Watchdog B102 probe (Spectrum Technologies, Aurora, IL, USA) in each of our six replicate alfalfa fields approximately one inch above ground level. Probes recorded these variables every 30 min throughout the 2012 season. Insect densities were measured with the aid of sweep net sampling performed on the same dates as aphid collection. We collected these insects using 30 sweeps of a fine-mesh net over the alfalfa canopy, doing this across multiple transects for every field. Collected insects were transferred to kill jars and stored in 95% ethanol until subsequent counting. In addition to counts of pea aphids (winged and unwinged), we also censused *A. ervi* parasitoids*,* ladybug predators (Coccinellidae), and potato leafhoppers (*Empoasca fabae*). The former two insects are large sources of pea aphid mortality that may be thwarted by facultative symbionts [98,99], while the latter is a potential competitor of the pea aphid, e.g., [100]. Insect counts, climate data, and mortality assay data (below) can be found in Table S2.

### 2.3. Mortality Assays

A subset of our beat-sampled pea aphids was brought back to the lab for mortality assays, with an emphasis on juvenile pea aphids ranging from the second to fourth instar stages. We reared these aphids on fava bean plants (*Vicia faba*) for eight days, and then recorded the numbers surviving, the numbers mummifying from *A. ervi* parasitoids, the numbers dying from apparent infections with *Pandora neoaphidis* (based on cadaver morphology), and the numbers disappeared or dead from unassignable causes.

### 2.4. Imputing Environmental Variables

On a handful of occasions, for particular fields, we were unable to measure one or more of the above environmental variables. We imputed these data using the MATLAB nearest neighbor method [101,102], helping to boost our statistical power. As reported by Smith and colleagues [60], results from this method showed good agreement with those from two other methods. Out of 84 possible time/field combinations (6 fields, 14 times), we estimated symbiont frequencies for 76 ($\bar{x}$ = 22.9 symbiont PCR-screened aphids, per time and field as mentioned above). Only 43 of the 760 possible environmental variable estimates (76 time/field combinations times 10 environmental variables) were imputed (see blue-shaded cells in Table S2).

### 2.5. Aphid Sampling across Overwintering Periods

In addition to our 2012 collections, we sampled pea aphids from the same six Berks County, Pennsylvania alfalfa fields across two overwintering periods, collecting in late October and again in May in the years 2012–2013 and 2013–2014 (Table S1). Aphids were collected using the same beat sampling and preservation methods described above. In between our collection points, pea aphid populations had experienced a single sexual generation, a small number of asexual generations, and an intervening period in which they overwintered as eggs.

### 2.6. Molecular Methods

We used diagnostic PCR to detect the presence/absence of facultative endosymbionts in individual aphids. Prior to DNA extraction we rinsed ethanol-preserved aphids once in a 6% bleach solution, and twice in distilled water. We then extracted DNA from single aphids, using previously published methods [82]. In brief, aphids were placed in 1.5 mL tubes, frozen with liquid nitrogen, then crushed with a pestle. We next added 200 µL of lysis buffer (0.1 M NaCl, 0.2 M sucrose, 0.1 Tris-HCl, 0.005 EDTA, and 0.05% sodium dodecyl sulfate) to each sample, before incubating for 30 min at 65 °C. Reactions were stopped with an addition of 66.6 µL of 8 M potassium acetate and a subsequent storage on ice for 40 min. We then centrifuged samples for 15 min, decanting the supernatant into a new 1.5 mL tube. Next, we pipetted 200 µL of 95% ethanol into these new tubes before centrifuging, again, for 15 min. We removed and discarded the supernatant, then washed the DNA pellet in 200 µL of 70% ethanol prior to centrifuging samples for an additional 15 min. The resulting supernatant was then discarded, and pellets were washed in 50 µL of ice-cold 100% ethanol prior to drying in a speed vac. Once dry, we suspended DNA pellets in 60 µL of low TE buffer, storing samples at −20 °C until further use.

Leveraging the ubiquity of the obligate symbiont *Buchnera aphidicola*, we assessed the suitability of each DNA extraction for facultative symbiont screening with a PCR targeting this bacterium’s 16S rRNA gene. As for *Hamiltonella* in our prior study [60], screening for the six focal facultative symbionts was also enacted through diagnostic PCR assays targeting 16S rRNA. Assays were developed in prior studies and have been shown to exhibit specificity for the symbionts in question. Protocols for these PCRs, and for DNA extractions, are described further within Table S3.

### 2.7. Statistical Analyses—Change over Time in 2012 and across Overwintering Periods

We conducted our statistics using R statistical software version 3.4.3 [103]. To begin, we assessed whether the frequencies of *Regiella*, *Serratia*, *Rickettsia*, *Rickettsiella*, and *Fukatsuia* changed significantly across the bi-weekly sampling intervals spanning 25 April 2012 to 25 October 2012. Toward this end, we used a mixed-effects generalized linear model (GzLM), with binomial error and a logit link function, running analyses separately for the six non-*Hamiltonella* facultative symbionts. The presence/absence status of each symbiont, in individual aphids, was used as a binary response variable for each model. Further, we used period (i.e., sampling timepoint) as our fixed effect and field as a random variable (Table S4).

For overwintering periods, we used the same statistical approach, giving insight into changes in symbiont frequency across cooler periods spanning October 2012—May 2013 and October 2013—May 2014 (Table S5).

### 2.8. Statistical Analyses—Environmental Correlates of 2012 Symbiont Dynamics—Simultaneous Analyses

We next tested which, if any, of our 10 measured environmental variables predicted the probability of presence/absence for the five symbiont species undergoing significant frequency shifts across the 2012 season. These dynamic symbionts included *Regiella*, *Serratia*, *Rickettsiella*, *Spiroplasma*, and *Rickettsia*. *Hamiltonella* was also dynamic, by this measure, but was not emphasized here due to its inclusion in our recent study [60]. *Fukatsuia* was the lone symbiont that did not change significantly in frequency across 2012. It was hence not included in statistical modeling designed to identify environmental correlates.

As stated above, the measured variables included: (1) counts of *A. ervi* parasitoids; (2) the proportion of pea aphids mummifying due to *A. ervi*; (3) counts of coccinellid beetle predators; (4) counts of alfalfa-damaging potato leafhoppers; (5) counts of pea aphids; (6) the proportion of pea aphids with wings as a measure of aphid stress; (7) the proportion of dead pea aphids exhibiting symptoms suggestive of *Pandora neoaphidis* fungal pathogen infection; (8) the proportion of pea aphids surviving the full 8-day rearing period; (9) vapor deficit, as a measure of aridity; and (10) temperature.

For counts, we calculated the average number of each focal insect found per 30 net sweeps across 3.4 replicate transects per time/field. We modeled temperature and vapor deficit as moving averages across the 10-day periods preceding each sampling date. Since we did not have climate data prior to 25 April, measurements for the first few days after this date were used to generate these climate averages for this first sampling point. Normalized values (*z*-scores) of these moving averages were used in our statistical models, which were computed from the mean and standard deviation. For insect counts and the proportions of winged aphids, we used log_10_ transformed versions of the data.

For each of the five identified dynamic symbionts we applied a mixed effects GzLM, with binomial error and a logit link function (Table S4). We included the 10 aforementioned variables as fixed effects, and field as a random effect. Single variables were removed from each starting model using the drop1 function of R. Reduced models were then compared to the prior best-fit “full” model based on Akaike Information Criterion (AIC) scores. We retained all variables whose removal worsened (i.e., increased) the AIC score by a magnitude 2 or more, while removing all others.

### 2.9. Statistical Analyses—Environmental Correlates of 2012 Symbiont Dynamics—Simultaneous + Lagged Analyses

Our prior publication documented instances where pea aphid populations exhibit detectable changes in *Hamiltonella* frequencies across 1–2 generations, suggesting the potential for symbiont-mediated responses to selection [60]. Given that the 13 separate 2-week intervals were traversed by our 2012 sampling, and the calculation of 16 pea aphid generations in this time, we estimate that about 1.2 pea aphid generations unfold within a single sampling interval. As such, one might expect adaptive symbiont responses to show either a correlation with simultaneous environmental variable measures—which were likely reflective of those in the days (and weeks) leading up to their collection—or measures occurring from the most recent sampling point, i.e., 2 weeks prior. In light of this, for all variables with *a priori* expectations of correlation with symbiont frequencies (i.e., based on prior lab work suggesting their modulation of symbiont fitness impacts or on prior field correlations), we examined not only the effects of environmental variables from the same time (time t), but also those from the prior time period (time t—2 weeks). We added these “lagged” variables to the previous “simultaneous” models, comprised of the simultaneously sampled variables (i.e., time = t for symbiont data and time = t for environmental variables) retained in our final models through the above-described drop1, AIC model comparison methodology. We then used this same drop1, AIC approach to determine if retention of any of these *a priori* predicted lagged variables improved our models (Table S4).

### 2.10. Hitchhiking Effects—Removal of Aphids with the Most Common Co-Infecting Symbiont Prior to Re-Assessing Symbiont vs. Environment Correlations

Based on our screening data, between 31.6% and 40.1% of PCR-screened pea aphids in 2012, 2013, and 2014 harbored two or more facultative symbionts (Table S1). Since co-infection was common, we considered whether some symbiont frequency shifts might not represent selection (dis)favoring aphids with the given symbiont, but selection acting on a common co-infecting symbiont, or on the co-infection and its resulting phenotype [93,104,105]. For this reason, we repeated the above statistical approach—i.e., GzLM modeling with drop1 analysis on simultaneous variables—for each dynamic symbiont in 2012 (i.e., all but *Fukatsuia*) after removing all aphids that harbored the focal symbiont and its most common co-infecting symbiont species (Table S5). For *Spiroplasma*, *Rickettsiella*, *Rickettsia*, and *Regiella*, the most common co-infecting symbiont across 2012 was *Hamiltonella*. For *Serratia* and *Hamiltonella* the most common co-infectors were, respectively, *Rickettsiella* and *Rickettsia*.

### 2.11. Hitchhiking Effects—Removing Co-Infected Aphids from Paired Timepoint Datasets, to Identify the Influence of Co-Infection on Symbiont Dynamics

To more precisely understand if temporal changes in symbiont prevalence were due to hitchhiking, we focused on frequency shifts across paired (i.e., adjacent) timepoints—i.e., May-to-October overwintering periods (Table S5), and 2-week spans in 2012. In doing so, we assessed significance in change over time for total symbiont frequencies and for focal symbionts without their most common co-infectors. Generalized linear mixed models (GzLM) were used toward this end. Adopting a similar approach to that used above, we assessed the AIC score of a model without time/period, for the given symbiont, before determining if the addition of time improved the AIC score by a value of 2 or more. 

To determine which symbionts to include in our full range of statistics, we identified all cases in which the frequencies of particular symbionts shifted by ≥20% across a 2-week period, showing strong parallelism among replicate fields. *Hamiltonella* met these criteria at times 3–4 (cool period), 4–5, and 6–7 (warm period) [60]. Also meeting these criteria was *Rickettsiella* at times 4–5, 5–6, 10–11, and 12–13 (Table S7). All such ≥20% symbiont shifts were found significant when GzLM statistics were performed on total symbiont frequency. For overwintering periods, our statistics revealed a range of significant symbiont shifts across the two seasons. We included all of these in below analyses regardless of magnitude.

We next generated smaller datasets for follow-up statistical analyses, addressing whether removal of all aphids with the focal symbiont and its most common co-infector eliminated the significance of the focal symbiont’s change across the given time. For these analyses, we first identified the most common co-infecting symbiont, for the focal bacterium, across each pair of timepoints chosen for analysis (see paragraph above). We then removed all aphids harboring both the focal symbiont and its most common co-infector from the dataset. Finally, we re-ran our above GzLM statistics, asking whether time substantially improved the AIC score relative to a model without this variable. In cases where the significance of the temporal shift was lost for the reduced, co-infection removed, dataset, we considered that the frequency shift stemmed either from hitchhiking or from selection on a phenotype conferred by a specific co-infection. Results of these analyses are deposited in Tables S5 and S7.

### 2.12. Hitchhiking Effects—Plotting Symbiont Species vs. Symbiont (Co-)Infection Context Trendlines for Comparisons to Hypothesized Patterns under Hitchhiking or Direct Selection

**Figure 1:**
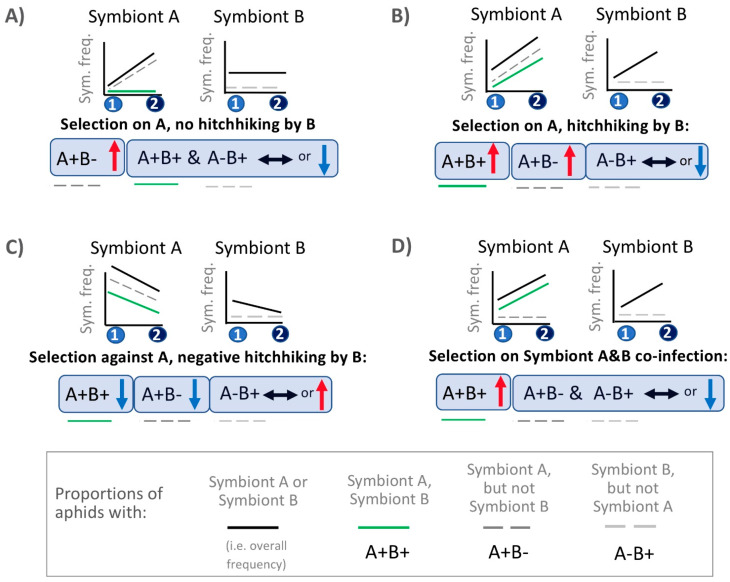
Hitchhiking or selection? Key for (co-)infection context deconstruction and its use in interpreting the symbiont dynamics shown in later figures. (**A**) Possible pattern expected if: there is positive selection on aphids with Symbiont A due to the symbiont’s fitness/phenotypic impacts, and if Symbiont B rarely lives with Symbiont A, showing, thus, no response via hitchhiking. (**B**,**C**) Patterns consistent with selection on aphids with one symbiont and hitchhiking by another. Under this scenario, selection acts on the phenotypic effects conferred by Symbiont A. This symbiont commonly lives with Symbiont B, which does not strongly impact Symbiont A’s fitness/phenotypic impacts, and which does not, itself, alter fitness at the given time. Symbiont B’s frequency dynamics, thus, extend from selection acting on its co-infecting Symbiont A partner. (**D**) Pattern expected if selection acts on a phenotype conferred due to the joint actions of two co-infecting symbionts. In this case, it is the co-infection that is under selection (only positive selection scenario shown for panel (**D**)—as done also for panel (**A**)). The boxed legend at the bottom decodes the line -color and -dashing schemes used to illustrate symbiont and (co-)infection context frequencies in (**A**–**D**). Note that y-axes represent the frequency of each symbiont or the particular (co-)infection type. Colored circles on the *x*-axis represent different sampling times.

In a separate set of analyses, we plotted symbiont species frequency and symbiont (co-)infection context frequency trendlines, comparing patterns with those in Figure 1 to draw further conclusions on the drivers of our symbiont dynamics. For this work we included all focal symbionts with ≥20% magnitude changes over 2-week periods in 2012. We additionally included all overwintering shifts exceeding 9% in magnitude. Trendlines were graphed to illustrate the overall frequencies of each focal symbiont (i.e., the symbiont exhibiting the large frequency shift—Symbiont A) and their most common co-infector (Symbiont B). In addition, we graphed the frequencies of aphids with the following (co-)infection contexts: Symbiont A+ Symbiont B+, Symbiont A+ Symbiont B−, and Symbiont A− Symbiont B+. We performed the below statistics on each of these same (co-)infection contexts as well.

Across the chosen paired timepoints, we performed GzLM statistics to assess whether the addition of time improved the AIC scores relative to that of base models—with only the random factor of field—in modeling symbiont or specific (co-)infection frequencies. We declared that particular symbiont species or (co-)infection contexts had changed in frequency over time when the AIC score was lowered by 2 or more upon the addition of time/period (Table S8). In examining these statistical outcomes, and the signs of our trendline slopes, our comparisons to patterns in Figure 1 assessed whether the data suggested: (i) Positive or negative selection on only Symbiont A with robust phenotypic effects across co-infection contexts, as partially exhibited in Figure 1A (i.e., the negative selection scenario is not shown). (ii) Positive selection on Symbiont A with hitchhiking by Symbiont B, as illustrated in Figure 1B. (iii) Negative selection on Symbiont A with negative hitchhiking by Symbiont B, shown in Figure 1C. (iv) Positive or negative selection on the co-infection, instead of selection acting on individual symbionts, as partially exhibited in Figure 1D (i.e., the negative selection scenario is not shown).

### 2.13. Hamiltonella-Focused Hitchhiking Effects—Microsatellite Genotyping to Ascertain Proliferation/Decline of Common Clones

A number of aphid species and populations have transitioned to permanently asexual reproduction, having lost the ability to produce sexual generations in response to seasonal cues [106,107]. In such aphids, linkage disequilibrium will be high among unlinked nuclear loci, and nuclear vs. mitochondrial loci. The lack of both recombination and independent assortment of heritable elements, will complicate efforts to pinpoint targets of selection, a problem extending to maternally inherited symbionts of such asexuals [108]. Pea aphids show a mixture of sexual and asexual reproduction throughout varying parts of their range, e.g., [109]. As such, portions of some populations may survive presumably mild winter seasons as asexual, parthenogenetic morphs, instead of overwintering as cold-tolerant eggs produced by matings between male and female sexual morphs. Indeed, within the currently studied Pennsylvania population we have resampled aphids with identical 6-locus microsatellite-genotypes across calendar years, suggesting some persistence of asexual clones [67]. Should such clones exhibit incidental differences in *Hamiltonella* frequency, then selection on clones for some non-symbiont conferred attribute could drive symbiont frequency shifts. 

To test this alternative to our hypothesis of direct selection on symbiont-conferred phenotypic variation, we performed microsatellite genotyping on 95 aphids from Times 6 (2 July, *n* = 51) and 7 (18 July, *n* = 41) of 2012, when *Hamiltonella* showed its greatest 2-week frequency increase (26.2%). In selecting aphids for genotyping, we aimed for a 50:50 split between *Hamiltonella* positive and negative aphids. Due to varying efficacy of microsatellite PCRs we came close to this ratio, with 59% of genotyped aphids from Time 6 harboring *Hamiltonella*, compared to 54.9% at Time 7. We genotyped these aphids at the six loci—S23, S24, ApH10M, APF08M, S30, and Aph08M—using a multiplex protocol and previously published primers described in Table S3 [67,110,111]. With these data we looked for the possibility that a common clone, or clones, with incidental tendencies toward high (or low) *Hamiltonella* infection might have proliferated (or declined) between Times 6 to 7. Assignment to particular common clones vs. singleton genotypes was performed on the basis of whether each 6-locus genotype was unique (singletons) or resampled (common clone). 

### 2.14. Hamiltonella-Focused Hitchhiking Effects—Buchnera ibpA Gene Promoter Genotyping

Given that *Hamiltonella*’s largest 2012 frequency rise unfolded across the 2012 summer’s hottest period (Times 6–7), we also considered whether shifts in this symbiont’s prevalence could have extended from selection on a co-infecting symbiont conferring positive or negative thermal tolerance properties. Since thermotolerance-conferring *Serratia* and *Fukatsuia* [89,92] were rare across the season, we focused on the obligate *Buchnera* symbiont. Prior work has shown that a 1 bp deletion creating a 12 bp stretch in *Buchnera’s ibpA* gene promoter causes high thermal sensitivity [96]. It has also been shown that facultative symbionts rarely live with such mutant *Buchnera* but are instead more common in aphids with the wildtype, 13 bp *ibpA* promoter [97]. It was thus possible that selection for thermally robust *Buchnera* with the wildtype *ibpA* allele, during the warm period of Times 6–7, could have driven up frequencies of facultative symbionts like *Hamiltonella*, through hitchhiking. To test this, we PCR-amplified and sequenced the *ibpA* locus from the *Buchnera* genome (Table S3) for 129 randomly selected aphids from times 6 (*n* = 61) and 7 (*n* = 69), assessing whether the spacer region of the promoter corresponded to the 12 bp or 13 bp allele. 

### 2.15. Presentation of Statistical Results

Due to the large volume of data analysis, we limited our in-text presentation of detailed statistical results for several of our focal analyses (i.e., symbiont frequency variation over time, correlations between symbiont frequencies and environmental variables—simultaneous analyses, symbiont frequency shifts across overwintering and 2-week frequency intervals). We placed most of these details, instead, in three tables cited in our Results section. For follow-up analyses (i.e., correlations between symbiont frequencies and environmental variables—time-lagged analyses, correlations between symbiont frequencies and environmental variables—robustness to hitchhiking effects), we present statistical outcomes at modest length in the text of the Results section, but full details can similarly be found in the below-cited supplementary tables.

## 3. Results

### 3.1. 2012 Longitudinal Field Study—Symbiont Frequency Variation over Time

**Figure 2:**
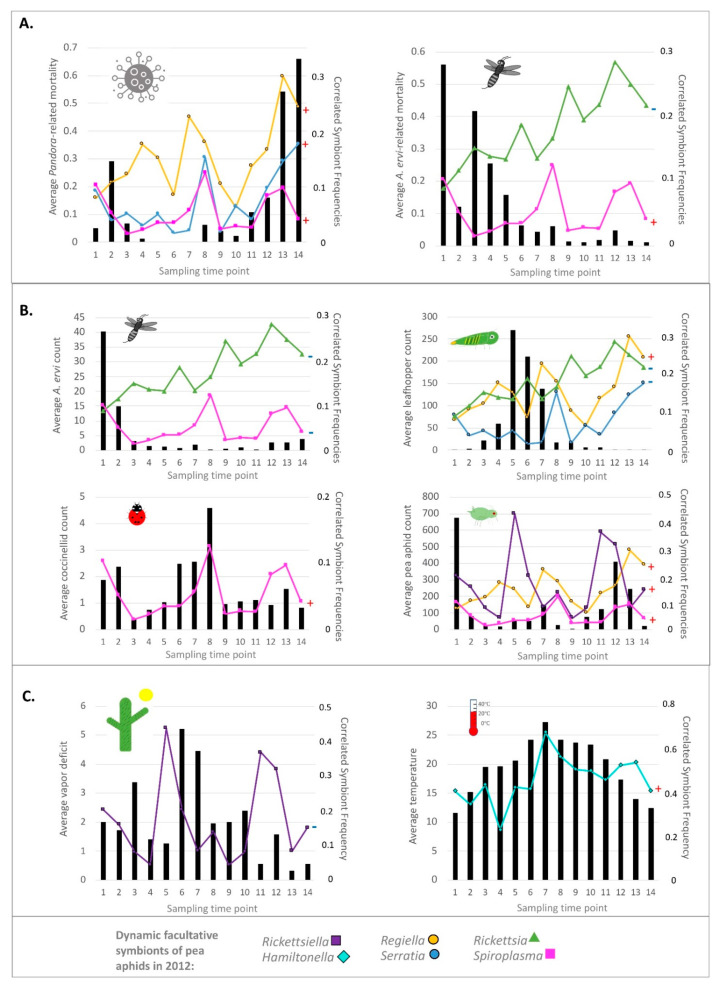
Correlations between the prevalence of facultative symbionts and environmental variables across the 2012 field season. Here we graph all significant results from our assessments of whether environmental variables (time t) predict symbiont frequency dynamics (time t), ascertained through diagnostic PCR. Each plot shows the data for the environmental variable (left *y*-axis) and prevalence/frequency of each symbiont (proportion of aphids infected) showing a correlation with the given variable (right *y*-axis). The directions of each correlation are denoted with a “−” or “+” to the right of each symbiont trendline. (**A**) Correlations with mortality due to *Aphidius ervi* parasitoids (ascertained by mummification) and *Pandora neoaphidis* fungal pathogens (based on fungal cadaver morphology). (**B**) Correlations with insects counted from our sweep net samples, including *A. ervi* parasitoids, an alfalfa-feeding competitor (potato leafhopper—*Empoasca fabae*), predatory coccinellid beetles, and pea aphid density. (**C**) Correlations with climatic variables, i.e., vapor deficit (aridity) and temperature (for *Hamiltonella*—based on data originally reported in Smith et al., 2021 [60]). For each symbiont we use colors and symbols consistent with those used in other figures. Not shown here are significant correlations with environmental variables without clear *a priori* expectations (Tables S9 and S10). Environmental variables were plotted as averages, as were symbiont frequencies, in contrast with other figures where frequencies were shown as pooled estimates. At each time point PCR screening data were obtained from an average of 126 aphids and 5.5 fields.

Frequencies of all symbionts, except for *Fukatsuia*, varied across 2012 according to our generalized linear mixed models (Table 1 and Table S4; Figure S1). *Hamiltonella* was the most prevalent, with an overall frequency of 46.2% when pooled across *n* = 1765 PCR-screened aphids from the six targeted fields and 14 sampled time points. As reported by Smith and colleagues [60], *Hamiltonella* reached a minimum frequency of 23.2% on 6 June (time 4), and a maximum of 68.1% on 18 July (time 6). Next in overall prevalence was *Rickettsia*, found in a pooled frequency of 18.2% of surveyed aphids (Figure S1). This alphaproteobacterial symbiont began the season at its minimum frequency of 9.3% (time 1—25 April). It then underwent a gradual, though non-monotonic, frequency increase throughout the season, reaching a pooled frequency peak of 29.0% on 15 August (time 9), and staying near or above 20% for remainder of the season. These trends differed only slightly when plotting averages from across fields (Figure 2), instead of pooled frequencies, with a peak of 28.5% coming on 26 September (time 12).

Across 2012, *Rickettsiella* exhibited the third highest prevalence, being found in 17.2% of PCR-screened aphids pooled across our study. Reaching a season-wide low of 4.3% on 6 June (time 4), this gammaproteobacterial symbiont hit its peak pooled frequency, of 43.7%, just 13 days later on 19 June (time 5). Found at lower abundances were *Regiella* (15.6% pooled frequency across 2012), *Serratia* (7.2%), *Spiroplasma* (5.2%), and *Fukatsuia* (4.8%). Pooled frequencies of the former three encompassed dynamic ranges of 23.2%, 15.9%, and 11.4%, when comparing season-wide maxima and minima.

### 3.2. Simultaneous Statistical Analyses of the 2012 Field Study—Symbiont Frequencies vs. Pandora Neoaphidis Pathogens 

Focusing on the five newly identified dynamic symbionts (i.e., all but the previously studied *Hamiltonella* and the non-dynamic *Fukatsuia*), we performed generalized linear mixed models to identify environmental factors correlating with symbiont frequencies. As described above, field was treated as a random effect. Fixed effects for each of our five models were obtained through drop1 analysis and AIC model score comparisons. Final models for each symbiont are presented in Table 2, and all significant results are highlighted in Figure 2. 

Among the environmental variables hypothesized to correlate with particular symbiont trajectories (Table S10), pressures from *Pandora neoaphidis* fungal pathogens were the most commonly predicted and the most commonly significant correlations. Specifically, with predicted anti-pathogen effects for *Regiella*, *Rickettsia*, *Rickettsiella*, and *Spiroplasma*, we found that the frequencies of *Regiella* (z-value: 5.845; *p*-value: 4.13 × 10^−8^) and *Spiroplasma* (z-value: 3.561; *p*-value: 3.69 × 10^−4^) showed positive correlations with the proportion of aphids dying from likely *Pandora* infections. While not correlating with *Pandora*-triggered mortality, *Rickettsiella* showed a negative correlation with vapor deficit (z-value: −4.326; *p*-value: 1.52 × 10^−5^). Viewed, conversely, as a positive correlation with the high humidity conditions known to favor fungal pathogens like *Pandora*, the dynamics for this symbiont were, thus, at least consistent with responses to pathogen pressures. Additionally, correlating with mortality from *Pandora* was the frequency of *Serratia* (z-value: 3.725; *p*-value: 1.96 × 10^−4^), a symbiont that—to our knowledge—has not been widely tested in assays against this pathogen. 

In assessing the negative result for the predicted anti-pathogen defender *Rickettsia*, we note that it reached its highest pooled prevalence mid-season (Figure S1). At this time, mortality from *Pandora* was still low, though beginning a trend of rising late-season prevalence (Figure 2A—left panel). *Rickettsia* remained prevalent throughout the remainder of the season, although it declined during the two times of highest *Pandora*-triggered mortality (times 13 & 14). Such patterns were not fully inconsistent with a *Rickettsia-*mediated selective response to this pathogen. But when coupled with the symbiont’s rarity of during an early season *Pandora* spike, they suggest, perhaps, a weaker relationship with *Pandora* pressures than those seen for *Regiella*, *Spiroplasma*, and *Serratia*.

### 3.3. Simultaneous Statistical Analyses of the 2012 Field Study—Symbiont Frequencies vs. Parasitoids, Temperature, and Coccinellid Beetles

Recent findings have implicated *Spiroplasma* as an anti-parasitoid defender, with resistance to *A. ervi* being encoded by a modest number of European strains [90,91]. And in our final model for this symbiont (Table 2), we found a positive correlation between its frequency and the proportion of aphids mummifying from *A. ervi* parasitism (z-value: 3.561; *p*-value: 0.02884). Paradoxically, *Spiroplasma* frequencies exhibited a negative correlation with the counts of *A. ervi* from our sweep-netting (z-value: −2.190; *p*-value: 0.02852). Graphical inspection revealed these two correlations to be among the weakest of those described above (Figure 2). In addition, the borderline significance of these relationships contrasted with that seen for most of the above-mentioned symbiont vs. *Pandora* correlations. This would seem to underscore the tentative nature of the *Spiroplasma* vs. *A. ervi* relationship.

Expectations of symbiont frequency correlations were less clear for coccinellid predators. For example, one previous study showed that *Hippodamia convergens* coccinellids experienced reduced survival when feeding on pea aphids harboring *Serratia* or *Hamiltonella* as larvae. Counteracting this potential benefit, however, was a finding from the same study in which surviving adult *H. convergens* that had fed on these symbiont-infected aphids had larger mass [99]. In a separate study [112], feeding on aphids with either *Serratia* or *Regiella* reduced survival of another coccinellid, *Harmonia axyridis*; but feeding on those with *Hamiltonella* did not. This study further found that *Regiella* prolonged times of *H. axyridis* development, suggesting some consistency to its effects [112].

It was posited that the harmful effects of symbionts that may consistently accrue for some predators (e.g., *H. axyridis*) could confer indirect benefits to the nearby siblings of consumed pea aphid clones. However, despite this expectation, we saw no correlation between *Regiella* and coccinellid predators, and only a marginally significant, negative correlation between coccinellids and *Serratia* (z-value: −1.947; *p*-value: 0.05158). *Spiroplasma* frequencies, in contrast, significantly tracked coccinellid sweep net counts (z-value: 3.997; *p*-value: 6.41 × 10^−5^), a finding of interest given the similarity of this bacterium to the male-killing *Spiroplasma* of *H. axyridis* [83].

In contrast to the above-described relationships, symbiont frequencies – aside from those previously reported for *Hamiltonella* [60] – did not correlate with temperature. This negative result unfolded despite modest *a priori* expectations. For example, an early report suggested positive effects of *Rickettsia* and *Serratia* on pea aphid fitness under warm temperatures, albeit in just one of three clonal backgrounds [88]. Subsequent studies demonstrated positive effects of *Serratia* on the survival and fecundity of multiple aphid clones after lab-exposure to heat shock conditions [74,89]. Follow-up field-based research provided further supporting evidence, suggesting positive impacts of *Serratia* on pea aphid population growth rates [113]. Despite this work, our present 2012 statistics provided no evidence that that temporal variation in *Serratia* prevalence was predicted by temperature—matching results from a prior, 2011-based study on the same pea aphid populations in Pennsylvania [64]. We similarly found no evidence for a relationship between temperature and *Rickettsia* under our simultaneous statistical analyses (Table S4). 

Beyond the thermal expectations for *Serratia* and *Hamiltonella* [60,73,74,75,82], *Regiella* had been contrastingly found to be beneficial under permissive-to-cool temperatures, and potentially harmful under warm conditions in one North American pea aphid clone [74,76]. Frequencies of this symbiont also showed a negative correlation in relation to temperature, when studied across pea aphid populations in Japan [114]. In spite of this, we found no significant relationship between temperature and *Regiella* frequency in our present 2012 Pennsylvania study (Table S4).

Among the remaining climatic variables with *a priori* expectations, we similarly found that aridity (vapor deficit) did not improve models predicting *Serratia* frequency when modeled as a simultaneous predictor. A positive correlation between *Serratia* and this variable had previously been demonstrated across a spatial context in a global study of pea aphids from numerous host races [115]. 

### 3.4. Time-Lagged Statistical Analyses of the 2012 Field Study—Comparing Symbiont Frequencies vs. a Priori Predicted Environmental Correlates Sampled 2-Weeks Prior

Since selective responses will unfold across spans of several generations, we examined the capacities for environmental factors from the prior sampling period (i.e., t-2 weeks—equivalent to 1.2 pea aphid generations, on average, in 2012) to predict frequencies of symbionts at time t. Focusing only on *a priori* hypothesized variables, we re-assessed negative results for the correlations expected between: *Serratia* vs. *A. ervi* parasitoids (based on an un-repeated finding, from 1 clone/strain [98]); *Rickettsia*, *Serratia*, and *Regiella* vs. temperature; *Regiella* vs. coccinellid predators; *Serratia* vs. vapor deficit; and both *Rickettsia* and *Rickettsiella* vs. *Pandora* pathogens. Given predicted relationships between *Regiella* and the development of wings in aphids [116], we further tested for a non-selective, plasticity-induced correlation between this symbiont vs. the proportion winged aphids.

In doing so, we retained all significant factors from our simultaneous generalized linear mixed models (Table 2) and added all lagged (*a priori*) variables predicted to correlate with prevalence for the given symbiont. As done for the simultaneous-factor-only models, we then implemented drop1 analysis and AIC model score comparisons, keeping lagged variables whose removal worsened AIC scores, and eliminating previously kept simultaneous variables if they did so as well. Results of the new, full models are presented alongside those of our simultaneous-variable-only models in Table S4, with the label “simultaneous + lagged”. They are further summarized in Tables S9 and S10. 

We found that addition of lagged variables improved the predictive power (i.e., lower AIC score) for models of several symbiont species. Notably, the addition of the 10-day moving average of vapor deficit, calculated for a time of t-2 weeks, significantly predicted *Serratia* frequencies at time t (z-value: 2.798; *p*-value: 0.00515), supporting expectations from Henry et al. 2013 [115]. Addition of both the 10-day moving average for temperature and the proportion of aphids dying from *Pandora* at times t-2 weeks improved the AIC score for the model of *Rickettsia* frequency relative to that of the base model with only the previously identified simultaneous variables. Each variable in the final simultaneous + lagged model for *Rickettsia* exhibited borderline statistical significance (z-value_temperature_: 2.368, *p*-value_temperature_: 0.01787; z-value*_Pandora_*: 1.96, *p*-value*_Pandora_*: 0.04966). 

Addition of *Pandora*-triggered mortality measured 2-weeks prior to symbiont frequency estimates, led to a slight improvement in the AIC score of the model for *Rickettsiella*. However, this lagged factor was negatively correlated with *Rickettsiella* prevalence, and showed only marginal significance (z-value: −1.807; *p*-value: 0.0707). Lagged models were not attempted for *Spiroplasma* since all variables with *a priori* predicted correlations showed significance in simultaneous-variable-only models. For *Regiella*, addition of lagged variables did not improve the AIC score beyond that of the base model, including only the originally significant simultaneous variables (Tables S4, S9 and S10).

### 3.5. Common Symbiont Co-Infections in Our 2012 Study

Facultative symbionts of pea aphids often live alongside other facultative symbiont species in the same aphid hosts, with frequent enrichment for particular species combinations [67,117]. We, hence, considered whether the dynamics of some focal symbiont might be driven by selection on co-infecting symbionts, and whether the above-discussed environmental variables might have, therefore, been spuriously correlated with the frequencies of such hitchhiking symbionts in 2012.

**Figure 3:**
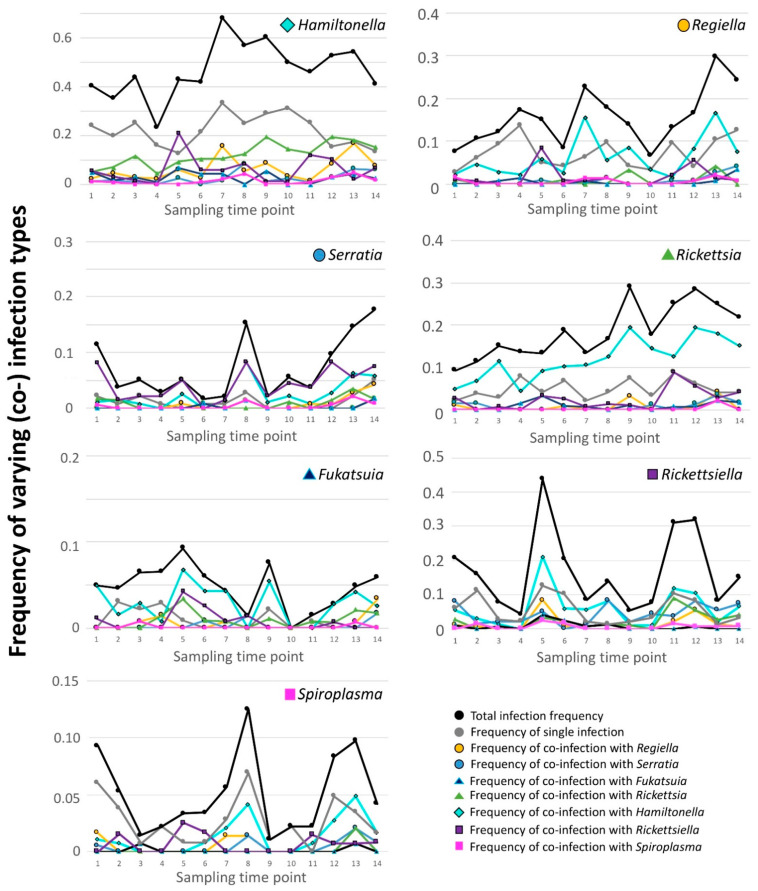
Co-infection contexts for facultative symbionts across the 2012 field season. Shown here are results of diagnostic PCR screening for each of seven facultative symbionts—in total and in relation to the presence of other symbionts in the same aphid hosts. All datapoints represent pooled frequencies across all sampled fields from the given time. Solid black lines represent total frequencies. Colored lines represent the frequencies of aphids harboring co-infections with the focal symbiont and other facultative symbionts. Gray lines represent the frequencies of the focal symbionts in aphids with no other co-infecting facultative symbionts (“single infections”). Several symbionts underwent large frequency shifts across paired time points, separated by only 2-weeks. These showed frequent consistency/parallels across replicate fields (Figure S1). Among these, shifts for *Hamiltonella* at times 3–4 and 6–7 were at least partially comprised of changes in the prevalence of single infections, arguing against the possibility that *Hamiltonella* hitchhiked in aphids with a second symbiont yielding a phenotype under stronger selection. Single infection trajectories were somewhat muted, but parallel to those of overall symbiont prevalence for the rapid frequency shifts of *Rickettsiella* at times 4–5, 5–6 (to a weaker extent), 10–11, and 12–13. In contrast, single infection frequencies of *Hamiltonella* at times 4–5 and *Regiella* at times 6–7 did not track overall frequencies of these two bacterial species. We follow up on these trends with more detailed (co-)infection context graphing in Figure 4. Note that, for the present figure, *y*-axis scales differ across the varying symbionts. Additionally, time points on the x-axis are separated by 2 weeks, spanning late April through late October.

Co-infection was indeed common in 2012, with facultative symbiont-bearing aphids harboring an average of 1.5 species when pooled across the season (Figure S2), a value close to totals observed in other studies [51]. Viewed from another perspective, 41.4% of aphids with a facultative symbiont harbored two or more such species. To further illustrate the co-infection phenomenon, we graphed the frequencies of aphids harboring single infections with a focal symbiont, next to the overall frequencies of aphids harboring this symbiont. Alongside these plots we also graphed the frequencies of aphids with the focal symbiont and any of the six possibly co-infecting facultative symbiont species (Figure 3).

In inspecting these graphs, we first observed that the overall dynamics for *Hamiltonella*—found previously to track temperature in 2012 [60]—seemed at least partially mirrored by dynamics of aphids harboring this symbiont alone (“single infection” upper-left panel of Figure 3). Single infections represented the most common (co-)infection context for *Hamiltonella* and comprised 21.8% of our *n* = 1765 aphids from 2012. The next most common (co)infection context for *Hamiltonella*-harboring aphids involved the presence of *Rickettsia*, with 11.7% of our surveyed aphids possessing these two symbionts. Although considerably rarer than single-infections, might the frequency of this co-infection suggest that hitchhiking with selectively (dis)favored *Rickettsia* could have driven some of our previously observed *Hamiltonella* dynamics? And might this, further, complicate the correlation between *Hamiltonella* and temperature?

Such potential for hitchhiking seemed even greater for several of the other dynamic symbionts. For three—*Serratia*, *Rickettsiella*, and *Rickettsia*—co-infections with their most common partner (*Rickettsiella*, *Hamiltonella*, and *Hamiltonella*, respectively) were more common than single infections (4.4% vs. 1.2%, 6.0% vs. 5.7%, and 11.7% vs. 4.8%). While single infection frequencies for *Regiella* and *Spiroplasma* (7.3% and 2.7%) exceeded the levels of co-infection with the most common co-infector for both, i.e., *Hamiltonella,* co-infections were still relatively common for these latter two symbionts (6.3% and 1.3%).

### 3.6. Simultaneous Statistical Analyses of the 2012 Field Study—Do Originally Significant Variables Remain in Our Models after Accounting for Hitchhiking?

We began our re-assessment of the above statistical findings (Table 1, Tables S4, S9 and S10) by creating new symbiont presence/absence datasets for *Hamiltonella* and the five symbionts found here to shift in frequency across 2012 (Table 1). For each new dataset we eliminated all aphids harboring the focal symbiont if they also harbored that symbiont’s most common co-infector for 2012. We then re-ran the above-described generalized linear mixed modeling approach, with drop1 analysis and AIC model score comparisons (Table S6).

Before attempting to identify environmental correlates for our newly adjusted symbiont data, we first assessed whether the addition of time (modeled as “period”) to base models with only a random factor of field led to significantly improved AIC scores. For *Rickettsia*, inclusion of period yielded a model AIC score of 263.31, which was deemed substantially worse than that of the base model—i.e., 257.75. This suggested that in aphids without *Hamiltonella*, *Rickettsia* did not change notably in frequency across the 2012 season, leading us to conclude that the 2012 dynamics for this symbiont were driven primarily by its co-infections with *Hamiltonella.* For this reason, the environmental correlates significantly predicting *Rickettsia* dynamics (i.e., negative correlations with *A. ervi* and potato leafhopper counts; a negative correlation with the proportion dying from *A. ervi* parasitism; Table S10), should be interpreted with caution. Indeed, rather than reflecting selection on variable fitness caused by *Rickettsia* presence/absence, correlations with particular environmental variables may instead have arisen due to fitness variability created from synergistic or additive effects of *Rickettsia*/*Hamiltonella* co-infections. It is also possible that *Rickettsia* was part of a longer-term hitchhiking trend, something possibly not examined here in an explicit fashion.

Given its lack of dynamics without its most common partner, we did not perform the follow-up statistics examining the relationship between environmental variables and *Rickettsia* prevalence after removal of common co-infections. In contrast to this case for *Rickettsia*, the inclusion of time lowered (i.e., improved) the AIC score by 2 or more for the other symbiont species, suggesting that they remained dynamic even after removal of all aphids harboring both the focal microbe and its most common co-infector. We thus proceeded with environmental model testing for these symbionts (see Tables S6 and S9 for a summary).

For *Regiella*, after beginning with all simultaneous variables, drop1 analyses and AIC score comparisons resulted in the following model: P(Reg)~stdmaVap + lgLeafHop + Ppandora + (1|fField). New to this model, compared to that obtained from the full dataset (which included the *Regiella*-*Hamiltonella* co-infections removed here), was vapor deficit, which exhibited a negative correlation with *Regiella* frequency (z-value: −2.427, *p*-value: 0.0152). Notably, *Pandora* remained significant, still exhibiting a positive correlation with *Regiella* (z-value: 2.119, *p*-value: 0.0340).

Starting with all simultaneous variables in our model, our drop1 analyses on the *Rickettsiella* dataset without *Rickettsiella-Hamiltonella* co-infections yielded the following as our final model: P(Rkla)~stdmaVap + lgAphDen + Psurv + (1|fField). While quantitatively different, this model was otherwise similar to the full, simultaneous model for this symbiont (Table 1). Most notably retained was the correlate of vapor deficit (z-value: −2.923, *p*-value: 0.00346), an inverse measure of humidity. As such, *Rickettsiella*’s positive correlations with a proxy for pathogen pressures appeared independent of co-infection with *Hamiltonella*—and hence, not likely a spurious extension of hitchhiking effects.

An analysis performed for *Spiroplasma*, after removing all aphids harboring co-infections between this Tenericute symbiont and *Hamiltonella*, yielded the following model: P(Spi)~lgAphDen + lgAervi + lgCoccin + Paervi + Ppandora + (1|fField). A priori predictions of correlations with *A. ervi* parasitoids and *Pandora* pathogens were, thus, robust to the presence or absence of *Hamiltonella*.

For *Serratia*, removal of aphids harboring this bacterium and its most common co-infector, *Rickettsiella*, led to the generation of the following model: P(Ser)~Psurv + Ppandora + (1|fField). Only the *Pandora* variable had been significant in our original model. Its sustained significance (z-value: 3.504, *p*-value: 4.64 × 10^−4^) suggested an intriguing relationship that was initially unexpected, and clearly not driven by co-infection with known anti-pathogen, and common *Serratia*-partner, *Rickettsiella*.

To follow up on the positive correlation with temperature reported for *Hamiltonella* in Smith et al. 2021, we removed two types of common *Hamiltonella*-centric co-infections from our models, in separate analyses. In the first case, a dataset lacking all aphids with *Hamitlonella* and *Rickettsiella* co-infections yielded a final model of P(Ham)~stdmaTA + lgWingAph + lgLeafHop + lgCoccin + (1|fField). Holdovers from our original model included both of the originally significant variables—the proportion of aphids with wings and temperature (z-value_temperature_: 4.054, *p*-value_temperature_: 5.04 × 10^−5^). New were counts of the potato leafhopper (z-value: −2.931, *p*-value: 3.37 × 10^−3^) and coccinellids (z-value: 2.051, *p*-value: 0.04029). In a separate set of analyses, we obtained this same model after removal of *Hamiltonella*-infected aphids that also harbored *Rickettsia*. Parameter estimates were slightly different, however, while leafhopper count became only marginally significant (z-value: −1.677, *p*-value: 0.09345). Across these two analyses, findings that the positive *Hamiltonella* vs. temperature relationship was independent of the symbiont’s co-infections with two common partners argued against a spurious, hitchhiking-driven environmental correlation. Discoveries of two potentially new predictors of this symbiont’s prevalence raised the possibility that co-infections with other symbionts could have obscured ecologically relevant, and possibly adaptive, correlations.

### 3.7. 2-Week Frequency Shifts in Our 2012 Field Study—Evidence for Hitchhiking?

Symbiont frequencies for *Hamiltonella* had been reported previously to swing substantially across 2-week periods between times 3–4, 4–5, and 6–7 [60], and the GzLM statistics applied here confirmed their statistical significance (Table 3 and Table S7). Additionally notable was our finding that other large frequency shifts, exceeding 20% in magnitude, characterized within-season changes for *Rickettsiella* (Figure S1 and Figure 2). Indeed, this symbiont’s spikes at times 4–5 and 10–11 represented significant changes, as did its declines at times 5–6 and 12–13. 

When we removed aphids harboring *Rickettsiella* and its most common co-infectors across the aforementioned dynamic timepoints (*Hamiltonella* at the first three; *Serratia* at times 12–13), GzLM reassessment revealed that the symbiont’s shifts over time remained significant (Table 3 and Table S7). This was true for *Hamiltonella* at times 3–4 and 6–7, which corresponded to two notable thermal events during the 2012 season [60]. However, at times 4–5, *Hamiltonella* dynamics were seemingly driven by co-infection with *Rickettsiella*, with evidence for change over time disappearing upon removal of all aphids with such co-infections from the dataset (p_full dataset_: 7.50 × 10^−4^; p_no *Rickettsiella* co-infections_: 0.3368). 

**Figure 4:**
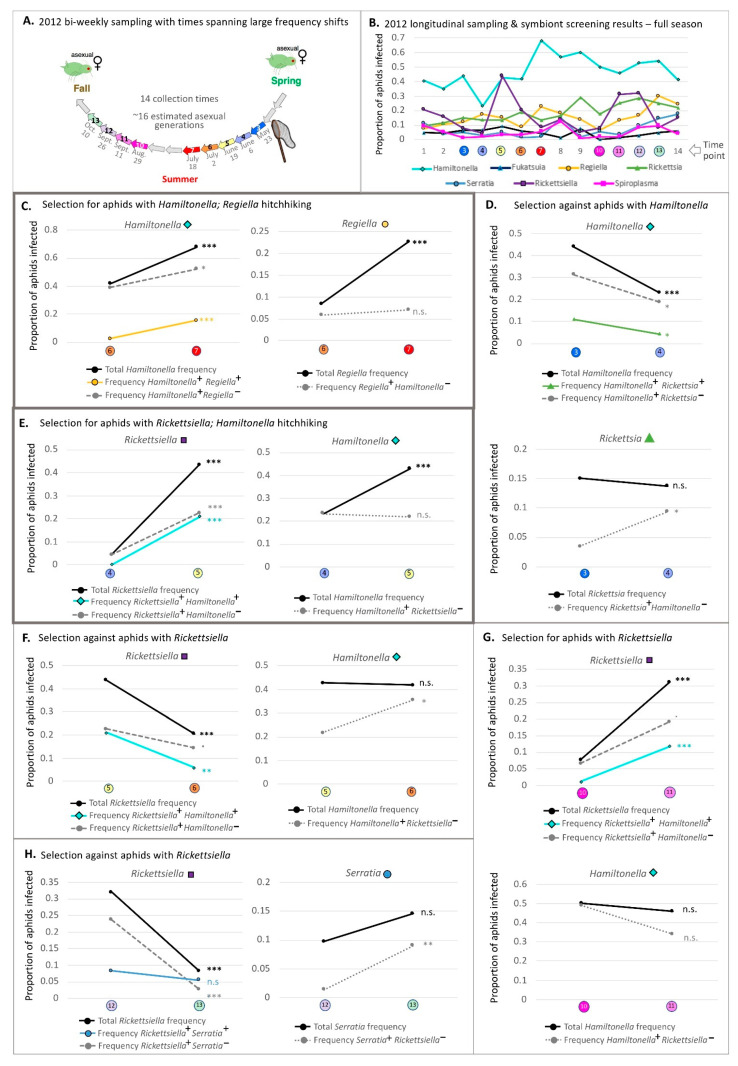
Direct selection vs. hitchhiking effects for *Hamiltonella*, *Rickettsiella*, and *Regiella* across 2-week time intervals in the 2012 field season. We limited our focus to symbionts undergoing frequency shifts ≥20% in magnitude across such time intervals. (**A**) Schematic of our 2012 field sampling, with colored arrows emphasizing the time points spanning 2-week intervals with large (≥20%) symbiont frequency shifts. (**B**) Re-graphing of data from Figure S1, to show—in one panel—the total frequencies, pooled across fields and (co-)infection contexts, for all seven facultative symbionts. (**C**–**H**) Shown here are symbiont and (co-)infection type frequencies across the focal 2-week intervals, pooled across all replicate fields. Conclusions drawn, using the logic of Figure 1 and the patterns/significance seen for the below-described trend-lines, are stated at the top of each panel. Black lines illustrate the overall prevalence of each symbiont undergoing a ≥20% magnitude shift (Symbiont A, left or top graph) or the frequency of its most commonly co-infecting symbiont (Symbiont B, right or bottom graph). Solid, colored lines (left or top graph) show the frequencies of aphids harboring both co-infecting symbionts (A+B+). Dashed gray lines show the proportions of aphids harboring the focal symbiont without this co-infector (A+B−, left or top graph) or the proportion of aphids with Symbiont B but not Symbiont A (B+A− right or bottom graph). Results of mixed effects GzLM statistics (with binomial error and the logit link function) are indicated to the right of symbiont frequency trendlines. These statistics compared two models, one with only field as a random effect and one that also included time. Significant results indicate that the model with time was significantly better, suggesting a shift in the frequency of the symbiont or (co-)infection type. Abbreviations are as follows: “***” 0 < *p* < 0.001; “**” 0.001 < *p* < 0.01; “*” 0.01 < *p* < 0.05; “.”; 0.05 < *p* < 0.1; n.s. = not significant. Statistics are presented in detail in Tables S7 and S8. Cases of hitchhiking are emphasized with bolded box borders. Note that times and colors of circles on the x-axes correspond to those indicated with the colored arrows in (**A**) and with the same colored circles in (**B**). *Y*-axes represent the frequency of each symbiont or the particular (co-)infection type (i.e., the proportion of all examined aphids harboring the symbiont or that combination of symbionts).

Focusing on these 2012 within-season dynamics, we graphed paired timepoint frequency trajectories for these two symbionts, and for their varying (co-)infection contexts—showing the trajectories and (co-)infection contexts for the focal species (Symbiont A) and its most common co-infector at the observed dynamic times. When comparing the resulting trendlines in Figure 4 to those of Figure 1, we saw evidence for a variety of patterns. Three pieces of evidence have led us to posit that many of these shifts are the result of selection rather than drift or changing modes/rates of symbiont transfer. First, the dynamics at these times for the focal symbionts showed strong parallelism across fields (Figure S1). Second, symbiont strains without a currently documented means of horizontal transmission, outside of parasitoids [118], proliferated at times when parasitoids were rare. Third, while evidence from the field suggests the potential for co-infection dependent impacts on rates of vertical symbiont transmission, prior modeling indicated that fluctuating transmission efficiencies were unlikely to cause several of the large and rapid frequency shifts seen in our studied population when adopting plausible estimates for field transmission (see [60] for a more detailed discussion of each point). 

With the benefits of GzLM statistical assessments, and AIC model score comparisons used to assess whether (co-)infection frequencies changed over (Table S8), we noted—for example—that the decrease in *Hamiltonella* at times 3–4 looked plausibly like selection against this symbiont, rather than: (1) hitchhiking with its most common co-infector (*Rickettsia*), or (2) selection against their co-infection (Figure 4D). Indeed, *Hamiltonella* frequencies declined significantly; the decline remained significant when examining all aphids with *Hamiltonella* that lacked *Rickettsia*; and the co-infection with *Rickettsia* showed a significant drop. Yet the overall frequencies of *Rickettsia* did not change, and the frequencies of this symbiont in aphids without *Hamiltonella* actually rose. 

At times 6–7 we reached a slightly different conclusion, noting how the pattern observed (Figure 4C) matched that of Figure 1B. Accompanying *Hamiltonella*’s overall frequency spike was an increase in the frequency of aphids with both this symbiont and *Regiella*. However, in addition, the frequency of *Hamiltonella* infected aphids that did not harbor *Regiella* increased as well. *Regiella*’s overall frequency rose by over 10%, but the frequency of aphids with *Regiella* but not *Hamiltonella* did not change significantly—barely budging above their starting frequency. The trends are consistent with selection acting on *Hamiltonella* infected aphids and with hitchhiking by *Regiella*. Indeed, examination of *Hamiltonella* single infections (without any other secondary symbionts; Figure 3) showed that they, too, spiked at time 7, just as frequencies of single *Hamiltonella* infections had declined across times 3–4.

At times 4–5 the most common co-infector of *Hamiltonella* was *Rickettsiella*. Like *Hamiltonella*, *Rickettsiella* frequencies rose drastically across this 2-week span, as did the frequencies of aphids with *Rickettsiella* but not *Hamiltonella*. However, frequencies of *Hamiltonella*^+^ *Rickettsiella*^—^ aphids did not change, suggesting that the *Hamiltonella* spike was a result of hitchhiking with *Rickettsiella*. 

It is interesting to note that vapor deficit had dropped considerably 2-weeks prior to the time 5 *Rickettsiella* spike, and that this aridity measure had risen drastically by time 6 (Figure 2C), when the symbiont’s frequency proceeded to plummet. This *Rickettsiella* decline did not appear to be a case of negative hitchhiking, nor of selection against a particular co-infection (Figure 4F vs. Figure 1). Intriguingly, *Rickettsiella*’s time 10–11 spike (Figure 4G vs. Figure 1A) was similarly deemed to have likely stemmed from direct selection on fitness variability created by this symbiont, and also corresponded to a notable decline in vapor deficit (Figure 2C). We saw no evidence for negative hitchhiking or selection against *Rickettsiella-Serratia* co-infections during *Rickettsiella*’s time 12–13 decline, which seemed less related to clear changes in aridity levels (Figure 2C,H).

### 3.8. Changes in Symbiont Frequency across Overwintering Periods

In furthering our goal of attempting to disentangle direct selection on particular symbionts from hitchhiking and from selection on co-infections, we report now on the trends of symbiont frequency across the 2012–2013 and 2013–2014 overwintering periods. Consistent declines across both periods had supported the idea that *Hamiltonella* is disfavored when it is cool, as had the steeper decline across the colder 2013–2014 period [60]. When removing all aphids with *Hamiltonella* and its most common 2013–2014 overwintering co-infector (*Rickettsiella*) from the dataset, we found that the second-year frequency decline was still evident (*p*-value: 4.73 × 10^−3^). However, with removal of aphids harboring *Hamiltonella* and its common co-infector across 2012–2013—*Rickettsia*—the marginally significant decline of *Hamiltonella* was erased (Table 3).

**Figure 5:**
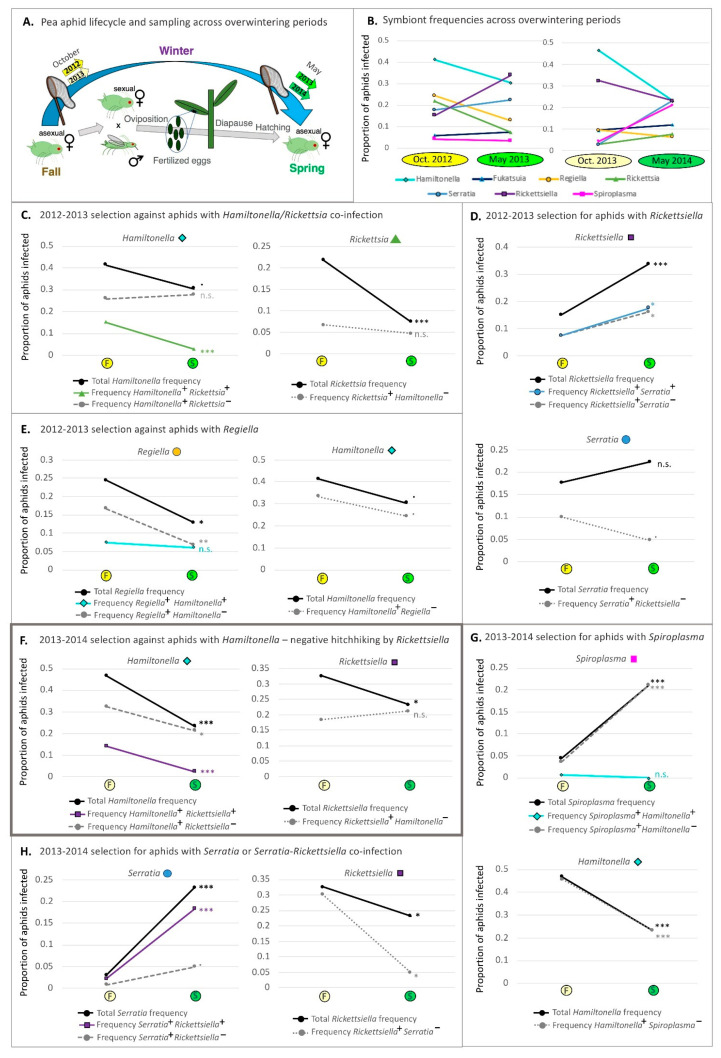
Direct selection vs. hitchhiking effects for facultative symbionts of pea aphids across overwintering periods. As shown in Smith et al. 2021, *Hamiltonella* frequencies declined across two overwintering periods. Illustrated here, in addition to *Hamiltonella*, are dynamics for the other six symbionts. Unlike *Hamiltonella*, none showed consistent effects across the 2012–2013 and 2013–2014 overwintering periods, although the frequency of co-infections between *Serratia* and *Rickettsiella* seemed to rise across both (panels **D**,**H**; see also Figure S3). (**A**) Sampling scheme across overwintering periods, in addition to a description of the pea aphid lifecycle, across these spans, for cyclically parthenogenetic aphids. Colored arrows indicate sampling times (in October and May) and correspond to the colored circles/ovals used in B-H (in which “F” = fall; “S” = spring). (**B**) Symbiont frequencies, inferred from diagnostic PCR, across the sampling points indicated in (**A**). (**C**–**H**) Graphs of overall symbiont frequencies (black lines) and frequencies of aphids with particular (co-)infection types (colored and dashed gray lines), as done in Figure 4. Conclusions are stated at the top of each panel. These were reached using the logic of Figure 1 and patterns of statistical significance associated with the below-described trend-lines. In short—each panel shows the overwintering frequency trajectory of a focal symbiont (Symbiont A) and its most common co-infecting symbiont (Symbiont B) from this time. Results of mixed effects GzLM statistics (with binomial error and the logit link function) are indicated to the right of symbiont frequency trendlines. These statistics compared two models, one with only field as a random effect and one that also included time. Significant results indicate that the model with time was significantly better, suggesting a shift in the frequency of the symbiont or (co-)infection type. Abbreviations are as follows: “***” 0 < *p* < 0.001; “**” 0.001 < *p* < 0.01; “*” 0.01 < *p* < 0.05; “.” 0.05 < *p* < 0.1; n.s. = not significant. Statistics are presented in detail in Tables S5 and S8. Unlike our approach for the 2-week frequency shifts in 2012 in which only symbionts undergoing ≥20% frequency shifts were treated as focal symbionts (Figure 4), we performed statistics and graphical illustrations for all symbionts undergoing a significant (or marginally significant) frequency shift of ≥9% across an overwintering period. Most of these symbionts were modeled as the ‘focal symbionts’ for graphing (except *Rickettsia*—panel (**C**)).

An inspection of (co-)infection frequency trends provided an explanation for this latter discovery (Figure 5C; Table S8). In particular, like those for *Hamiltonella*, overall frequencies of *Rickettsia* had also declined across this period, a decline that was evident for aphids with both this symbiont and co-infecting *Hamiltonella*. However, there was no change in the prevalence of *Rickettsia*^+^ *Hamiltonella*^—^ aphids nor in that of aphids with the *Hamiltonella*^+^ *Rickettsia*^—^ infection context. We reasoned that the pattern was consistent with selection against the *Hamiltonella/Rickettsia* co-infection based on logic similar to that used in Figure 1D.

Across the subsequent 2013–2014 overwintering period, (co-)infection context trendline trajectories and associated statistics (Figure 5E; Table S8) suggested that the *Hamiltonella* overwintering decline was a likely function of selection against this symbiont. However, a corresponding decline in its most common co-infector, *Rickettsiella*, without a decline in *Rickettsiella*^+^ *Hamiltonella*^—^ aphids suggested negative hitchhiking by this latter symbiont (see similar hypothetical trend in Figure 1C). GzLM statistics, and AIC model score assessments, on a dataset without *Hamiltonella*^+^ *Rickettsiella*^+^ aphids supported these results (Table 3)—with retention of the significant *Hamiltonella* decline in this smaller dataset (*p*-value: 4.73 × 10^−3^), and the loss of significance for *Rickettsiella* (*p*-value: 0.892).

While other symbionts showed significant shifts in frequency across overwintering periods (Figure 5 and Figure S3; Table 3, Tables S5 and S8), no other species was consistent in its trends across both seasons. We do note, however, that the proportions of aphids with the *Serratia–Rickettsiella* double infection rose significantly across both years (e.g., Figure 5D,H; Table S8). In 2012–2013, proportions went from 7.5% in October to 17.6% in May. For 2013–2014 they rose from 2.2% to 18.3%. These symbionts have been found to commonly co-infect aphids from multiple populations in the United States [67]. Such trends, thus, raise the possibility for undefined, seasonal, benefits arising specifically from their co-infection.

### 3.9. Hamiltonella-Focused Hitchhiking Effects—Aphid Microsatellite Genotyping and Buchnera ibpA Gene Promoter Genotyping

Through our above focus on facultative symbiont co-infections, it has become plausible to argue that symbiont dynamics can be driven by selection on aphids targeting: (1) the phenotypic effects of individual symbionts and, separately, (2) the joint phenotypic effects particular co-infections. The third means of rapid, parallel symbiont trajectories in our studied populations appears to involve hitchhiking, in which selection on the effects of one facultative symbiont seems to drive the spread of passenger co-infectors. 

But, of course, facultative symbionts comprise just one source of heritable genetic variation in a pea aphid population. Given the extensive degree of variation encoded by the pea aphid genome, and its impacts on a range of ecologically relevant traits [119,120,121], it is clear that natural selection is acting on other heritable targets in the field, with a similar potential to drive rapid evolutionary change [122,123]. Such selection may, in turn, drive hitchhiking effects for facultative symbionts.

To dissect this potential, we focused on *Hamiltonella* during its time of greatest increase, across the 2012 season’s hottest period—spanning 2 July to 18 July (i.e., times 6–7; Smith et al., 2021). Through the above analyses, we have seemingly ruled out hitchhiking with another facultative symbiont as the explanation for *Hamiltonella* spread during this time. However, while we have posited adaptive spread of *Hamiltonella* due to its beneficial effects under warm temperatures [73,74], and while several indicators suggested strong thermal stress to pea aphids in the field at this time (e.g., the average temperature from each 4, 5, 6, and 7 July exceeded the upper temperature threshold for pea aphid development—i.e., 28 °C as cited in [124]), it remains possible that selection targeted some other heritable driver of thermal tolerance, or some other non-thermal phenotypic attribute showing an incidental association with *Hamiltonella.*

**Figure 6:**
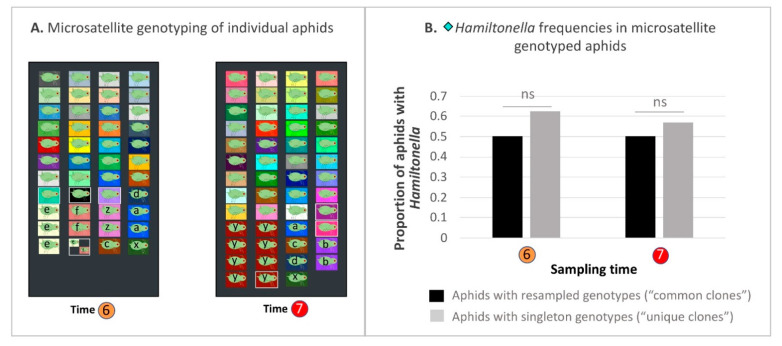
No evidence for *Hamiltonella* hitchhiking with favored aphid clones at times 6–7 during our 2012 field season. Analyses performed to further assess whether *Hamiltonella*’s rise in frequency during a hot time period (times 6–7; Smith et al., 2021 [60]) was a product of hitchhiking. In brief, genotyping of pea aphids at six microsatellite loci provided no support for selection on common aphid clones with incidentally high *Hamiltonella* infection rates. (**A**) Infographic gives a unique color to each unique genotype for the *n* = 44 and *n* = 51 microsatellite-genotyped aphids from times 6 and 7. Aphids with resampled genotypes are labeled with letters (a, b, c, d, e, f, x, y, z). Boxes outlined in white represent aphids with only 5 genotyped loci. For these aphids we inferred a plausible 6-locus genotype assignment by declaring the aphid to most likely encode a singleton genotype (i.e., if it was not identical to any one aphid at all 5 of its genotyped loci) or to more likely belong to a resampled genotype/clone (i.e., if it was identical to one or more resampled genotypes at all 5 of its genotyped loci). For one aphid (Time 6), the genotype appeared equally likely to assign to genotype/clone “e” or “f”. (**B**) Frequencies of *Hamiltonella* in microsatellite genotyped aphids from times 6–7. If selection was operating on common clones with high propensities for *Hamiltonella* infection one would expect aphids with resampled genotypes to have a higher prevalence of *Hamiltonella* than those with unique genotypes. Yet, Fisher’s Exact Test statistics revealed that they did not (*p* > 0.05 for the comparisons at each time, indicated with the “n.s.” abbreviation, for not significant).

We first considered the potential for selection on pea aphid clones with incidentally high *Hamiltonella* frequencies. Through our microsatellite genotyping of aphids collected at times 6–7, we obtained six locus genotypes for *n* = 84 aphids, and five locus genotypes for *n* = 8 individuals (Table S11). After imputing full genotypes for these partially genotyped individuals, we found that 69 aphids had singleton genotypes that were found only once in the dataset. The remaining 26 encoded resampled genotypes (Figure 6A). The most common genotype (genotype “y”) was sampled from *n* = 8 aphids, which had all been collected at time 7. No other genotype was sampled from more than *n* = 3 or *n* = 4 aphids. *Hamiltonella* was found in four of the eight genotype y individuals, and three of the six other aphids with resampled genotypes at time 7—with an infection frequency estimate of 50%. It was comparably found in 56.8% of *n* = 37 aphids with singleton genotypes from this time (Figure 6B). At time 6, *Hamiltonella* was found in 62.5% of *n* = 32 aphids with singleton genotypes and in 50% of *n* = 12 aphids with resampled genotypes.

To summarize, genotyping results revealed no strong clonal structure to our population, making it less likely that one or a few favored clones with incidental *Hamiltonella* infection drove this symbiont’s proliferation. Furthermore, findings of similar *Hamiltonella* frequencies in aphids with common vs. rare genotypes similarly argued against the likelihood of such a hitchhiking effect. It remains possible, however, that *Hamiltonella* was more subtly correlated with aphid genotype, and that selection on the associated cryptic genetic variation (e.g., within the aphid nuclear genome) drove the observed trends. While it will be difficult to rule out this form of hitchhiking, an investigation of this possibility remains necessary in our broader effort to establish whether *Hamiltonella* is indeed a direct target of thermally mediated selection. 

In our second test of an alternative, hitchhiking-centered hypothesis, we considered the possibility for selection on phenotypic variation created by *Buchnera* [21]. We tested one such possibility by focusing on the likely phenotypic impacts of allelic variation in this symbiont’s *ibpA* gene promoter. In particular, aphids with strains of *Buchnera* encoding the 13 bp spacer at this locus show higher thermal tolerance and an increased tendency to associate with facultative symbionts, compared to those with the 12 bp spacer [96,97]. Might selection on aphids with more thermally tolerant *Buchnera* across times 6-7 have enabled incidental spread of *Hamiltonella* due to a linked increase in hospitality to facultative symbionts?

To address this question, we genotyped 129 aphids at the *ibpA* gene promoter (Table S1), attempting to ascertain whether the spread of *Hamiltonella* at Times 6–7 could have arisen due to selection on the wildtype 13 bp spacer variant (Figure S4a). We discovered, however, that all aphids genotyped at time 6 harbored the 13 bp spacer length, as did all of those genotyped at time 7 (Figure S4b). There was thus no evidence for *Hamiltonella* hitchhiking with favored *Buchnera* variants. The rarity of the 12 bp spacer variant was consistent with prior field surveys [97], and it remains possible that more common, unexplored allelic variation in *Buchnera* could have been the target of selection. 

## 4. Discussion

### 4.1. Signals of Symbiont-Mediated, Seasonal Pea Aphid Adaptation

Prior research has illustrated how seasonal variation in the environment can drive rapid adaptation in multivoltine organisms [122,125]. Furthermore, work in systems with facultative, vertically inherited microbes has shown the potential for rapid, symbiont-mediated responses to selection in the field [126]. Motivated by such findings, our research here revealed that all but one facultative symbiont of the pea aphid shifted in frequency across a single growing season in a southeastern Pennsylvania population of the pea aphid’s alfalfa host race. 

Changes in symbiont frequencies were consistent with their potential significance in helping aphids to meet seasonal demands from a fluctuating environment. Accordingly, the frequencies of some symbionts were correlated with pressures known to mediate their costs and benefits in the lab. For instance, one such lab-predicted impact—anti-pathogen defense for *Regiella*—was supported by the correlations seen for this symbiont and the rates of pea aphid mortality from *Pandora* pathogens across the 2012 season. Field evidence for such protective services was obtained previously in Europe, when two pea aphid clones were found less likely to succumb to fungal pathogens when harboring this symbiont [79]. But in that study, *Regiella* appeared to lower the overall survival of such aphids after field rearing, raising the question as to whether anti-pathogen services are enough to confer a net benefit. If the *Regiella* vs. *Pandora* correlation seen here (Figure 2A) was driven by pathogen pressures, it would suggest that, indeed, they sometimes are.

While our analyses reveal a number of other correlations worthy of future study (e.g., Table 2; Figure 1), one of our primary motivations was to test *a priori*, lab-derived hypotheses, like the one predicted for *Regiella* and *Pandora* (Table S10). Leveraging an improved study design, relative to the one adopted for a 2011 study of this same pea aphid population [64], the greater numbers of field replicates and shortened intervals between sampling applied here were reasoned to increase our power to detect such correlations. But in spite of this, several hypotheses were not supported, including the expected correlation between *Serratia symbiotica* and changing seasonal temperature [74,89]. 

In addition to the above-mentioned (1) *Regiella* vs. *Pandora* correlation, the *a priori* symbiont vs. environment hypotheses that were supported, and robust after hitchhiking assessments, were the positive correlations between: (2) *Spiroplasma* vs. *Pandora* pathogen-triggered mortality, and (3) *Hamiltonella* vs. temperature. A positive correlation between (4) *Rickettsiella* vs. humidity was also consistent with a lab-based prediction of anti-fungal defense [86]. Finally, (5) the tentative correlations between *Spiroplasma* prevalence vs. *A. ervi* parasitoids were consistent with recent discoveries of this symbiont’s anti-parasitoid effects [90,91]. The fact that symbiont frequencies rose at the same times as these biotic and abiotic pressures, and that they were often lower at other times, supports a model in which seasonally useful ecological services and alternating net symbiont costs alter facultative symbiont prevalence across a single season [70]. 

In follow-up studies it will be important to relate symbiont frequencies to alternative measures of such environmental pressures. Temperature, for instance, has been found to be variably influential in studies examining maximum or minimum temperatures, estimated separately across daytime and nighttime periods [127]. The importance of these separate measures was seen in a study on heritable *Cardinium* symbiont prevalence in *Culicoides* biting midges, when examined across geography in Israel [128]. Among our several detected correlations, more detailed examination will, thus, at least be useful in dissecting the relationship between *Hamiltonella* and temperature.

In considering alternative hypotheses, it is conceivable that selection was not the primary, or sole, driving force of symbiont dynamics and that phenomena including horizontal symbiont transfer could have also, or instead, shaped our findings. However, we do not yet find strong cause to invoke this mode of transfer as a driver of large, rapid pea aphid symbiont dynamics. Indeed, while work in other aphids suggests the likelihood for such movement [84,118], research on some pea aphid symbionts (*Serratia*, *Hamiltonella*, *Rickettsia*) has not uncovered frequent movement through such routes [129,130]. Nevertheless, recent discoveries of plant-mediated movement for slightly divergent *Serratia* symbionts in other aphids [131], suggest a need for more accurate estimates on the rates of horizontal transfer, and for a broader effort to identify the avenues through which it occurs. This is made all the more evident when we note that such transmission has been rarely examined, to our knowledge, for a majority of pea aphids’ symbiont species (i.e., *Spiroplasma*, *Fukatsuia*, *Regiella*, and *Rickettsiella*).

Beyond the potential for seasonal pulses of horizontal transfer, it is also conceivable that seasonal variation in vertical transmission efficiency could explain the observed facultative symbiont dynamics. While this possibility has not yet been documented for pea aphids [67], evidence for an effect of temperature on symbiont transfer in other aphids, and in insects beyond, supports this possibility [20,132]. Prior modeling, however, did not support the idea that changing rates of vertical transmission could be the sole driver of our facultative symbiont dynamics. In particular, fluctuations between the varying levels of vertical transmission deemed plausible from prior field estimates [67] appeared unable to explain the speed of large-magnitude *Hamiltonella* shifts first reported in a recent study [60]. 

### 4.2. Past, Present, and Future Studies on Selection Targets and the Impacts of Co-Infection

Multi-species communities of facultative symbionts are common in insects, e.g., [50,133,134], including the pea aphid [67]. To improve our understanding of symbiont-mediated insect adaptation and the resulting symbiont dynamics in natural populations [135], further research is needed on the generalities extending from such co-infections [134]. Are symbiont-conferred host-level phenotypes altered when a second or third facultative symbiont is present? Can symbiont effects be additive or synergistic, and could some symbionts even suppress the beneficial effects of their co-residents? 

For now, our field evidence suggests the answer to the first question is often yes, as large-magnitude symbiont frequency shifts are often comprised of parallel change across multiple (co-)infection contexts (Figure 3 and Figure S3). Indeed, in nine of the twelve cases examined for 2-week or overwintering period frequency change, the focal symbiont showed parallel trends under both single infection and co-infection contexts (Figure 4 and Figure 5). 

More direct answers to these questions have emerged from the lab. For example, it was shown that anti-pathogen phenotypes of *Rickettsia* and *Spiroplasma* were robust to the addition of an additional facultative symbiont species [136]. More recently demonstrated was the finding that various combinations of *Regiella*, *Rickettsiella*, *Spiroplasma*, and *Hamiltonella* did not induce major alterations to individual symbionts’ anti-pathogen or anti-parasitoid services seen, originally, under single infection [105]. This suggests that multi-species co-infections may continue to provide the conditions necessary for individual symbionts to fulfill their ecological services. 

In contrast to these discoveries, however, were findings that a co-infection between *Serratia* and a particular *Hamiltonella* strain (“strain D”, [60]) induced strong costs and modified anti-parasitoid defensive phenotypes, with the latter quite possibly being a direct result of low aphid fitness [137]. A similar cost induced by a related *Hamiltonella* strain, realized under single infection and when paired with *Regiella*, may have lowered survival benefits conferred normally by this latter symbiont after pathogen challenge [104]. In a more recent study, it has also been shown that the impacts of co-infection may depend on the identity of the infecting symbiont strain, rather than the identity of the symbiont species [93]. This discovery suggests the importance of strain-level screening in future field-focused efforts. While more laborious, such efforts may prove valuable given the additional importance of strain variability in shaping anti-pathogen, anti-parasitoid, and thermal phenotypes [74,138,139].

Looking beyond the pea aphid system, it has occasionally been seen that symbiont phenotypes are retained under co-infection in other host insects. For example, *Spiroplasma*’s fecundity restoration phenotype in *Drosophila neotestacea* remains robust in flies also harboring non-defensive *Wolbachia* symbionts [23]. Similarly, suppression of the survival of two *Leptopilina* parasitoid species was of similar magnitude in *Drosophila melanogaster* harboring either *Spiroplasma* alone or a *Spiroplasma* and *Wolbachia* co-infection [140]. In contrast to these results, the strength of *Wolbachia*’s cytoplasmic-incompatibility phenotype—expressed under single infection, by individual strains—can be lessened under particular co-infection contexts [141]. Since symbiont phenotypes are often shaped by symbiont density, the abilities of heritable symbiotic bacteria to alter the densities of co-infecting symbionts [142,143,144] suggest a wider, unexamined, potential for the unexpected modification of symbiont phenotypes under co-infection.

### 4.3. Selection on Specific Co-Infections as Exceptions to the Rule

To summarize, while it is clear that there are exceptions in systems with numerous symbiont species like the pea aphid, generalities have begun to emerge from our study. In particular, given that most of the observed frequency trends (e.g., Figure 4 and Figure 5) mirrored the patterns predicted for a subset of the selective scenarios (Figure 1A–C), the data from our studied pea aphid population appear to suggest that symbiont-encoded phenotypes are functional across co-infection contexts in the field. 

Our alternative discovery, that co-infections may sometimes be the direct target of selection (e.g., patterns matching Figure 1D), suggests a series of alternative possibilities. Seen for two of the twelve examined symbiont frequency shifts (Figure 4 and Figure 5), we reasoned that, for one such case, a *Hamiltonella–Rickettsia* co-infection may have been synergistically costly between October 2012 and May of 2013. For the second, we concluded that a *Serratia–Rickettsiella* co-infection was synergistically beneficial across the October-to-May span of 2013–2014. In each case, trajectories of the involved symbionts under single infection were either: (1) not significantly dynamic, or (2) dynamic in directions that counterbalanced the direction of the co-infection shift. 

The idea of synergistic symbiont effects raises a range of interesting questions. To our knowledge, there are few examples of how a single selective pressure would favor co-infection, making it possible that different selection pressures acting simultaneously were responsible for these observed trends. However, one prior plausibility does come to mind. Twelve years ago, it was proposed that the loss of *Hamiltonella*-mediated anti-parasitoid defense at warm temperatures was rescued by co-infecting *Fukatsuia* symbionts [145]. While follow-up work, done in a more controlled fashion, did not detect a defense-restoring phenotype for a single strain of *Fukatsuia* [71], the example—still plausibly true—illustrates how symbiont-driven solutions to an environmental pressure may work only under certain contexts, and that co-infecting symbionts could expand this range of contexts by buffering the sensitivities of their co-infecting partners. If so, then it is possible that a single selective force could indeed favor co-infections under a subset of seasonal conditions. 

In examining why selection may act against a particular co-infection, but not the constituent symbionts individually, it is helpful to consider cases of demonstrated physiological cost. One of the first suggestions that particular co-infections may be synergistically costly in the pea aphid system came from an earlier-described study by Oliver and colleagues [137], in which *Hamiltonella* and *Serratia* strains without large detriments under single infection became strongly detrimental when living together. Under co-infection *Serratia* proliferated to considerably higher titers, suggesting their behavior as the source of symbiont virulence, and indicating how symbiont interactions can be specific to co-infection contexts. Such synergistic costs do not yet appear to be the norm for the pea aphid system [e.g., 94,104,105]. While this fits with the apparent rarity of selection against particular co-infections in our present study, such costly co-infections might never become common enough in the field for the detection of such a phenomenon. In line with this, findings that some symbiont species pairings are rarer than expected across pea aphid populations [67], suggest some natural relevance of costly synergism. 

It should be noted that our focus on paired timepoints for dissections of (co-)infection context trajectories (e.g., 2-week intervals with ≥20% frequency shifts in 2012, and overwintering periods with significant shifts ≥9%) limits abilities to assess the modes of selection acting on individual symbionts or communities. Toward this end it is also worth noting that the *Hamiltonella–Rickettsia* co-infection that was seemingly disfavored across the 2012–2013 overwintering period had previously shown a gradual, fairly consistent increase across the 2012 growing season (Figure 3). Single infections with each symbiont did not show such a trend. Therefore, it remains possible that there was a subtle selective advantage to harboring both symbionts through their synergistic effects on host fitness and phenotypes, and that this was reversed under the cooler overwintering period. It remains to be seen whether such phenomena are repeatable across years. However, an examination of data from aphids collected from nearby fields, in 2011, suggests a similar potential for selection on the *Hamiltonella–Rickettsia* co-infection across later portions of the 2011 growing season (Figure S5; data from [64]). 

These possibilities, clearly, warrant further study. In such work particular attention should be paid toward co-infections enriched in American populations, including the *Serratia–Rickettsiella* and *Hamiltonella–Rickettsia* co-infections mentioned here, along with a common co-infection between *Hamiltonella* and *Fukatsuia*. While variably enriched across populations, these pairings have each been seen to be more common than expected by chance in a substantial proportion of the examined times and places, suggesting the potential for important interactions [67]. It was previously argued that these symbionts may improve each other’s transmission [67], although evidence under controlled conditions is still needed to test this possibility. And it is, furthermore, still plausible that these partnerships have synergistically beneficial, or unusually benign, effects on host phenotypes.

### 4.4. Hitchhiking among Maternally Inherited Elements 

Moving back to the realm of selection on individual symbionts, we return for a brief, final discussion of hitchhiking. Importantly, while many of our conclusions on symbiont vs. environment correlations were deemed robust to hitchhiking (Figure 6 and Figure S4, Table S10), this phenomenon does seem to occur in the pea aphid system, in at least some forms (i.e., compare bold-boxed examples in Figure 4 and Figure 5 to Figure 1B,C). As such, some facultative symbionts—imposing neither strong costs nor benefits; nor clearly impacting the services of their co-infectors—will occasionally find their frequencies altered by virtue of who they live with, and when such co-infectors become useful (or costly).

In this study we used a conservative definition to detect this form of hitchhiking, requiring: (1) that two commonly co-infecting symbiont species undergo a simultaneous, significant frequency shift in the same direction; in addition to (2) (co-)infection context trends matching those of Figure 1B or Figure 1C. For this reason, it is fully plausible that the phenomenon is more common than we have documented here. Indeed, a more subtle negative hitchhiking event may have unfolded across times 5–6, in the 2012 season (Figure 4F). At this time, apparent selection against *Rickettsiella* may have dragged down the frequency of *Hamiltonella*, due to their common association from the prior time period. However, countervailing selection favoring *Hamiltonella* when living without *Rickettsiella* (see dashed gray line in right portion of Figure 4F) may have balanced this source of *Hamiltonella* decline, leading to a case in which *Hamiltonella* did not change in frequency, overall, across this timespan. 

This example, and other possible instances, may have obscured or weakened correlations with particular environmental variables, or even created spurious correlations. While this will need to further be explored, it is important to note that many of our initially significant environmental correlates from 2012 (e.g., Figure 2) retained their significance after accounting for hitchhiking effects (Table S6; see also our Graphical Abstract). Furthermore, few variables without significance in our full models gained significance after re-running statistics without aphids harboring the most common co-infections. These trends were especially true for *Hamiltonella*, *Rickettsiella*, and *Spiroplasma*, and, still, to a modest degree for *Regiella* and *Serratia* (Table S10).

While hitchhiking between facultative symbionts may, hence, be of modest influence, other forms of hitchhiking are plausible in the pea aphid system. Tested but not supported here was selection on thermally tolerant *Buchnera* variants, and on common aphid clones, as vehicles for hitchhiking-mediated *Hamiltonella* spread (Figure 6 and Figure S4). Not explored here, though quite likely, would be hitchhiking between facultative symbionts and another maternally inherited element—mtDNA. Indeed, across many insects, mtDNA haplotypes go along for the ride while favored or manipulative symbionts spread through host populations [146]. The strength and existence of associations between mtDNA haplotypes and symbiont infection will depend on the recency of symbiont spread, the efficiency of the symbiont’s vertical transfer, and the rates of horizontal symbiont transfer [23,61,77,147,148,149]—factors that will similarly influence associations between different facultative symbionts [117]. Future explorations of facultative symbiont associations with aphid mtDNA will yield insight into the broader implications of symbiosis for genetic diversity and rates of evolution in host mtDNA genomes [150]. Similar studies on facultative symbionts and *Buchnera* should prove similarly illuminating in a broader effort to clarify how highly dynamic facultative symbioses shape more ingrained, obligate fixtures of host insect biology.

## Figures and Tables

**Table 1:** Generalized linear mixed models statistics assessing whether seven facultative symbionts changed in frequency over time (a.k.a. “period”) across the 2012 field season.

Symbiont Tested Across 2012	AIC in Model with vs. without Period ^†^	*p*-Value Representing the Difference between the 2 Models ^††^
*Fukatsuia*	231.59 vs. 230.16	0.0264 **
*Hamiltonella* *	408.2 vs. 471.4	1.99 × 10^−13^
*Regiella*	328.87 vs. 359.89	1.78 × 10^−^^7^
*Rickettsia*	352.20 vs. 370.78	2.46 × 10^−5^
*Rickettsiella*	351.05 vs. 485.35	2.20 × 10^−16^
*Serratia*	263.67 vs. 300.53	1.61 × 10^−8^
*Spiroplasma*	244.46 vs. 255.79	3.68 × 10^−4^

* Data from Smith et al., 2021 [60]. ** The only model in which the AIC was worsened by the inclusion of period. ^†^ Base model (without period) included only field, modeled as a random effect. The model with the lower AIC score is the superior model. ^††^ *p* < 0.05 indicates that the model with lower AIC is significantly better.

**Table 2:** Generalized linear mixed models identifying environmental correlates of symbiont frequencies across the growing season of 2012 (simultaneous analyses).

Symbiont	Final Model ^†^	Parameter Estimates and Associated Statistics ^††^				
			Estimate	Std. Error	z Value	Pr(>|z|)
*Regiella*	P(Reg)~lgAphDen + lgLeafHop + Ppandora + (1|fField)	(Intercept)	−2.6827	0.26804	−10.009	<2.0 × 10^−16^
		lgAphDen	0.2366	0.11165	2.119	0.0341
		lgLeafHop	0.32962	0.08362	3.942	8.08 × 10^−5^
		*Ppandora*	*1.76821*	*0.32237*	*5.485*	*4.13 × 10^−8^*
*Rickettsia*	P(Rick)~lgAervi + lgLeafHop + Paervi + (1|fField)	(Intercept)	−1.18695	0.10071	−11.786	<2.0 × 10^−16^
		lgAervi	−0.22329	0.11341	−1.969	0.04897
		lgLeafHop	−0.20100	0.07654	−2.626	0.00863
		Paervi	−1.12356	0.43613	−2.576	0.00999
*Rickettsiella*	P(Rkla)~stdmaVap + lgAphDen + Psurv + (1|fField)	(Intercept)	−2.79286	0.27472	−10.166	<2.0 × 10^−16^
		stdmaVap	−0.33461	0.07735	−4.326	1.52 × 10^−5^
		lgAphDen	0.40121	0.10271	3.906	9.37 × 10^−5^
		Psurv	1.01093	0.24990	4.045	5.23 × 10^−5^
*Serratia*	P(Ser)~lgLeafHop + lgCoccin + Ppandora + (1|fField)	(Intercept)	−2.6756	0.1743	−15.351	<2.0 × 10^−16^
		lgLeafHop	−0.3763	0.1052	−3.577	3.48 × 10^−4^
		lgCoccin	−0.3640	0.1870	−1.947	0.05158
		Ppandora	1.4043	0.3770	3.725	1.96 × 10^−4^
*Spiroplasma*	P(Spi)~lgAphDen + lgAervi + lgCoccin + Paervi + Ppandora + (1|fField)	(Intercept)	−5.0718	0.4726	−10.733	<2.0 × 10^−16^
		lgAphDen	0.7999	0.2033	3.934	8.34 × 10^−5^
		*lgAervi*	−*0.4758*	*0.2173*	−*2.190*	*0.02852*
		lgCoccin	1.0285	0.2573	3.997	6.41 × 10^−5^
		*Paervi*	*1.8833*	*0.8617*	*2.186*	*0.02884*
		*Ppandora*	*2.0677*	*0.5807*	*3.561*	*3.69 × 10^−4^*

^†^ Variable abbreviations are as follows: stdmaVap—vapor deficit; lgAphDen—pea aphid counts; lgAervi—*Aphidius ervi* parasitoid counts; lgCoccin—coccinellid beetle predator counts; lgLeafHop;—potato leafhopper counts; Psurv—the proportion of aphids surviving 8 days after field collection; Paervi—the proportion of aphids mummifying from *A. ervi* parasitism; Ppandora—the proportion of aphids dying from an apparent *Pandora neoaphidis* fungal infection; (1 |fField)—replicate alfalfa field (modeled as a random effect; all other variables are fixed effects). ^††^ last column: *p*-value = Pr(>|z|); significant variables with *a priori* expectations for the given symbiont are indicated in italics.

**Table 3:** Symbiont frequency shifts across 2-week intervals and overwintering periods and robustness to hitchhiking effects—i.e., does the result remain significant after removing all aphids with the most common co-infection (last column)?

Focal Symbiont	Time (2-Week Time Interval or Month Overwintering pd.)	Change in Focal Symbiont Prevalence	*p*-Value for Prevalence Change ^†^	Most Common Partner	*p*-Value for Prevalence Change after Removing Co-Infections w/Common Partner
*Hamiltonella*	3–4	−0.207	**2.32 × 10^−4^**	*Rickettsia*	**2.61 × 10^−3^**
	4–5	0.197	**7.50 × 10^−4^**	*Rickettsiella*	0.3368
	6–7	0.262	**2.62 × 10^−5^**	*Regiella*	**1.12 × 10^−3^**
	Overwintering: 2012–2013	−0.108	0.0673 ^††^	*Rickettsia*	0.9433
	Overwintering: 2013–2014	−0.234	**3.81 × 10^−5 ††^**	*Rickettsiella*	**4.73 × 10^−3^**
*Rickettsiella*	4–5	0.393	**7.04 × 10^−13^**	*Hamiltonella*	**2.42 × 10^−6^**
	5–6	−0.232	**8.35 × 10^−4^**	*Hamiltonella*	**0.0154**
	10–11	0.301	**3.92 × 10^−4^**	*Hamiltonella*	**0.0433**
	12–13	−0.236	**2.91 × 10^−7^**	*Serratia*	**3.18 × 10^−5^**
	Overwintering: 2012–2013	0.187	**4.82 × 10^−4^**	*Serratia*	**4.13 × 10^−5^**
	Overwintering: 2013–2014	−0.094	**0.0183**	*Serratia*	**9.93 × 10^−8^**
				*Hamiltonella*	0.892
				*Serratia* + *Hamiltonella*	**6.20 × 10^−5^**
*Regiella*	Overwintering: 2012–2013	−0.115	**0.0286**	*Hamiltonella*	**4.27 × 10^−3^**
*Rickettsia*	Overwintering: 2012–2013	−0.144	**8.96 × 10^−3^**	*Hamiltonella*	0.3869
	Overwintering: 2013–2014	0.048	0.0987	*Hamiltonella*	0.0862
*Serratia*	Overwintering: 2013–2014	0.203	**2.82 × 10^−8^**	*Rickettsiella*	**0.0113**
*Spiroplasma*	Overwintering: 2013–2014	0.167	**6.80 × 10^−6^**	*Hamiltonella*	**2.96 × 10^−6^**

^†^ Data shown only for shifts with marginal (0.1 > *p* > 0.05) or full (*p* < 0.05) significance (bold) for overwintering periods or for those symbionts with a frequency shift rounding up to 20% or more across a 2-week time interval in 2012. ^††^ Data from Smith et al., 2021 [60].

## Data Availability

All data generated in association with this study have been made available in the form of supplementary tables published online with this article.

## Supplementary Materials

The following are available online at https://www.mdpi.com/article/10.3390/insects12090805/s1, Figure S1: Facultative symbiont frequencies over time, graphed for each replicate alfalfa field, across our 2012 field season; Figure S2: Average number of facultative symbionts in infected pea aphids, across 14 sampling times in 2012; Figure S3: Co-infection contexts for facultative symbionts across two overwintering periods; Figure S4: The absence of a thermally sensitive *ibpA* promoter allele in obligate *Buchnera* symbionts across times 6–7, argues against selection on the thermal properties of this obligate symbiont as the driver of a large *Hamiltonella* shift; Figure S5: Data from 2011 collections in nearby southeastern Pennsylvania alfalfa fields reveal potential selection on the *Hamiltonella-Rickettsia* co-infection in the later periods of the growing season; Table S1: Aphid collection data, symbiont PCR screening, and *Buchnera ibpA* promoter genotyping results from our 2012 longitudinal field survey; Table S2: Environmental variables from our 2012 longitudinal field study, averaged by time and field, used in our statistical analyses; Table S3: Cycling conditions, primer sequences, and cocktail recipes for PCRs used for diagnostic symbiont screening, *Buchnera ibpA* gene promoter genotyping, and aphid microsatellite genotyping; Table S4: Statistical analyses on symbiont data from our 2012 longitudinal field study—symbionts vs. time and symbionts vs. environmental variables; Table S5: Statistical analyses on symbiont data from 2012-2013 and 2013-2014 overwintering study, including datasets in which co-infections with top partners were removed (i.e. hitchhiking assessments); Table S6: Statistical analyses on our 2012 longitudinal field study—symbionts vs. environmental variables with tests of robustness to hitchhiking effects (i.e. by model-reassessment after removing the most common co-infection); Table S7: Statistics on 2-week frequency shifts during 2012 longitudinal study, with an assessment of hitchhiking effects; Table S8: Statistics on 2-week and overwintering symbiont frequency shifts for deconstructed (co-)infection contexts; Table S9: Statistical analyses on symbiont data from our 2012 longitudinal field study—final/summarized statistical models from Tables S4 and S6; Table S10: Illustration of *a priori* hypotheses in relation to summaries of season-wide 2012 statistics (Tables S4 and S6), including simultaneous, time-lagged, and hitchhiking-removal analyses; Table S11: Microsatellite data generated for aphids from our 2012 longitudinal field study at times 6–7.
