# Supplementary material for: Frequent Drivers, Occasional Passengers: Signals of Symbiont-Driven Seasonal Adaptation and Hitchhiking in the Pea Aphid, Acyrthosiphon pisum
Source: Insects. 2021 Sep 8;12(9):805. doi: 10.3390/insects12090805 (PMC8466206; doi:10.3390/insects12090805)
Supplement: Supplementary file 1 [file insects-12-00805-s001.zip › Supplementary/Supplementary.pdf]

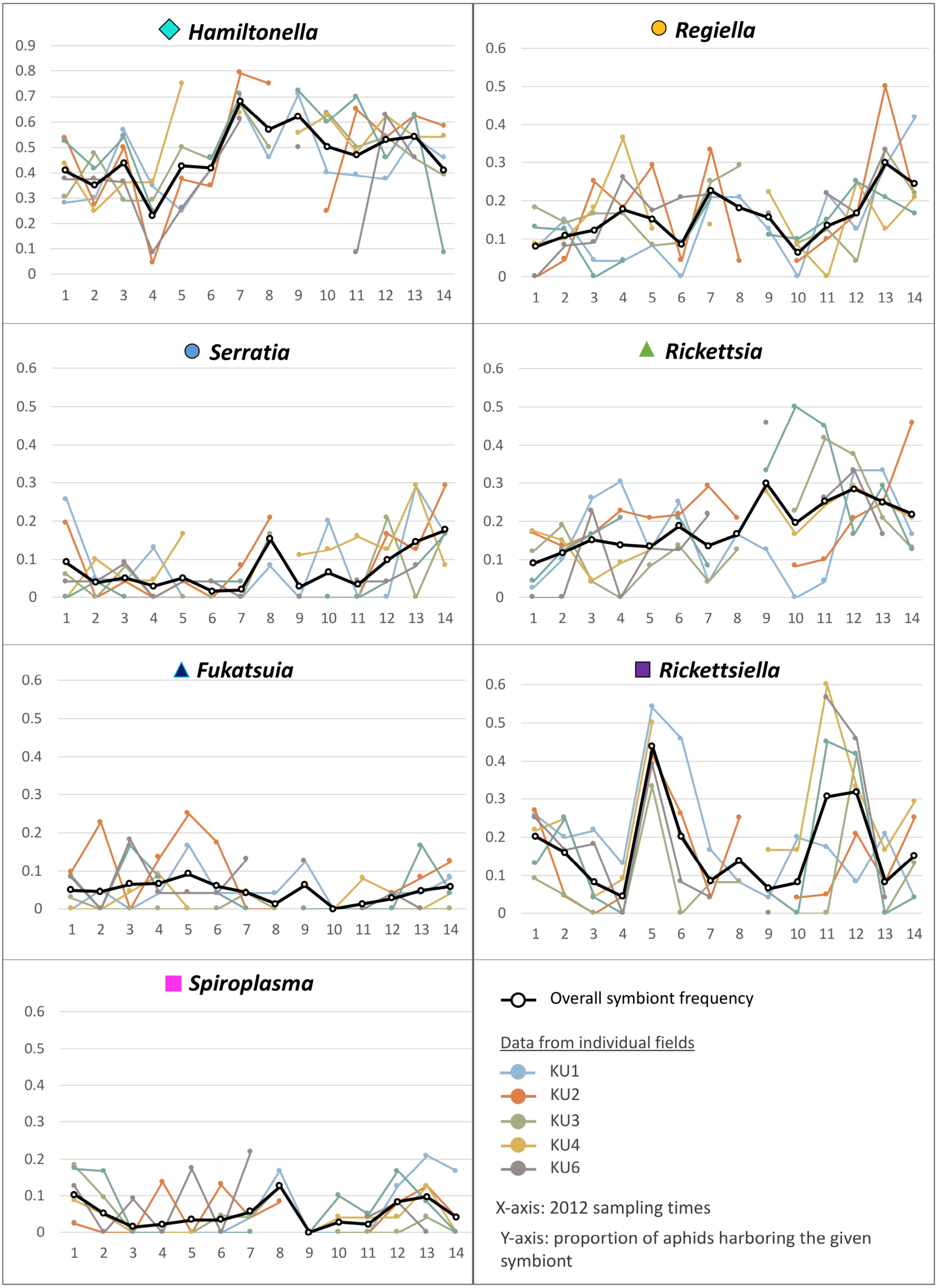

Hamiltonella
Regiella
Serratia
Rickettsia
Fukatsuia
Rickettsiella
Spiroplasma
Overall symbiont frequency
Data from individual fields
KU1
KU2
KU3
KU4
KU6
X-axis: 2012 sampling times
Y-axis: proportion of aphids harboring the given symbiont

**Figure S1: Facultative symbiont frequencies over time, graphed for each replicate alfalfa field, across our 2012 field season.** Data points represent frequency in each field. Black lines, with open white circles, give the average frequency across all sampled fields from a given time.

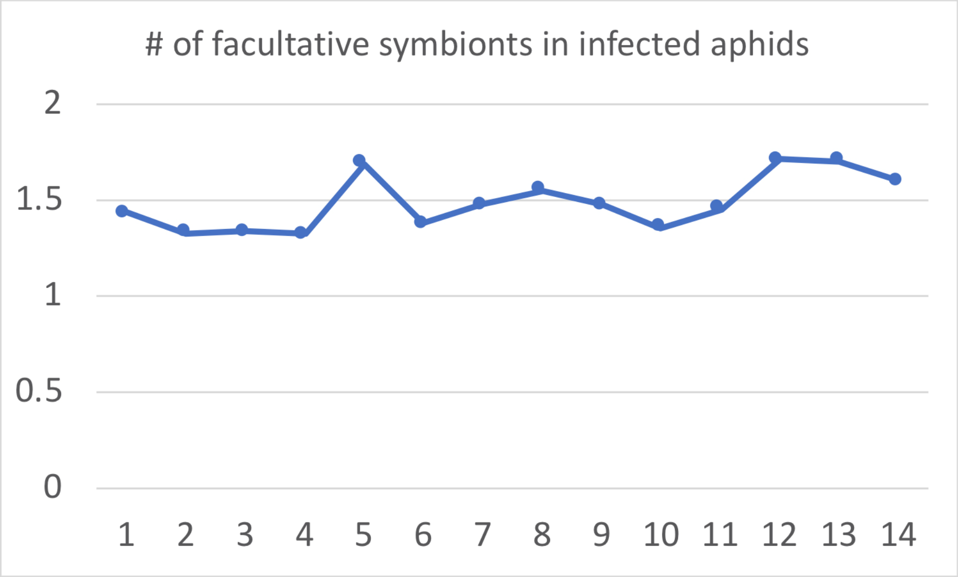


**Figure S2: Average number of facultative symbionts in infected pea aphids, across 14 sampling times in 2012.** Y-axis = # of symbionts per aphid. X-axis = sampling times, separated by 2-week intervals.

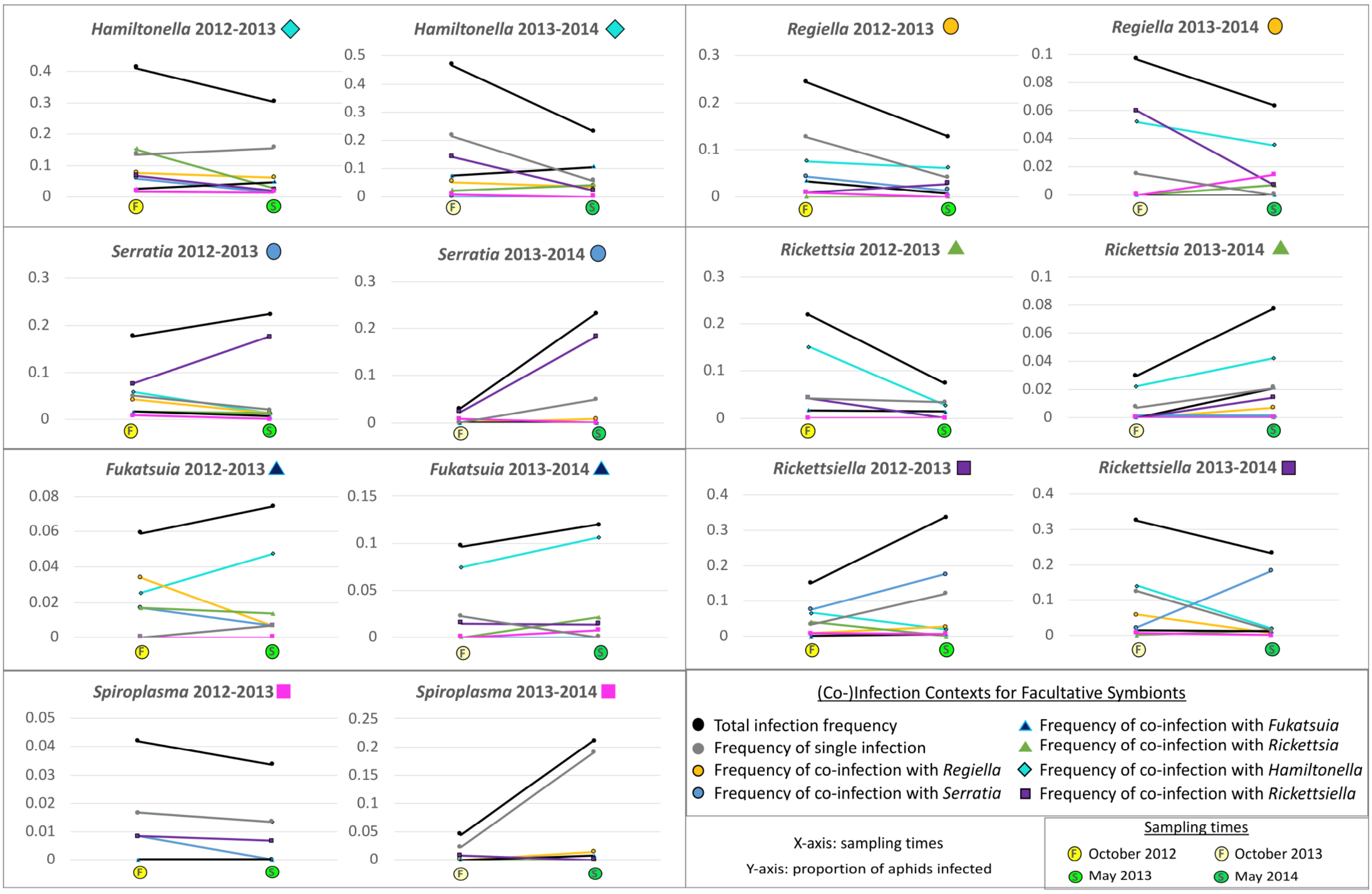

**Figure S3: Co-infection contexts for facultative symbionts across two overwintering periods.** Shown here are results of diagnostic PCR screening for each of seven facultative symbionts – in total (pooled across fields – black lines) and in relation to the presence of other symbionts in the same aphid hosts (colored and gray lines). Y-axis: proportions of aphids harboring the particular symbiont species, or those exhibiting the specific (co-)infection type. Note, that for the present figure, y-axis scales differ across the varying symbionts. Also, time points on the x-axis are separated by 2 weeks, spanning late April through late October. We use the same color scheme for fall ("F") and spring ("S") collections adopted for **Fig. 5**.

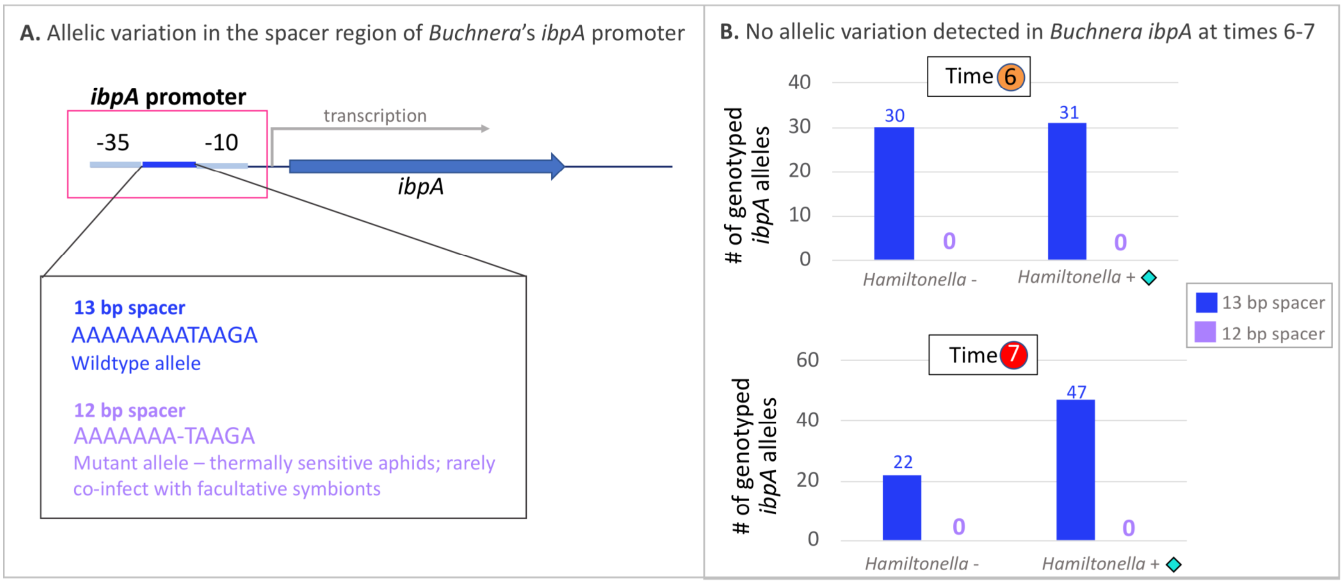


**Figure S4: The absence of a thermally sensitive *ibpA* promoter allele in obligate *Buchnera* symbionts across times 6-7, argues against selection on the thermal properties of this obligate symbiont as the driver of a large *Hamiltonella* shift**. Analyses performed to further assess whether *Hamiltonella*'s rise in frequency during a hot time period in 2012 (times 6-7; Smith et al. 2021) was a product of hitchhiking. A) *Buchnera*'s *ibpA* gene promoter region shows allelic variation in some pea aphid populations. Aphids harboring *Buchnera* with the wildtype (13 bp) variant have greater thermal tolerance than those with the mutant (12 bp) variant, and also greater tendencies to live with facultative symbionts like *Hamiltonella*. B) But genotyping of *Buchnera* from n=61 and n=69 aphids at Times 6 and 7 uncovered only the wild type allele.

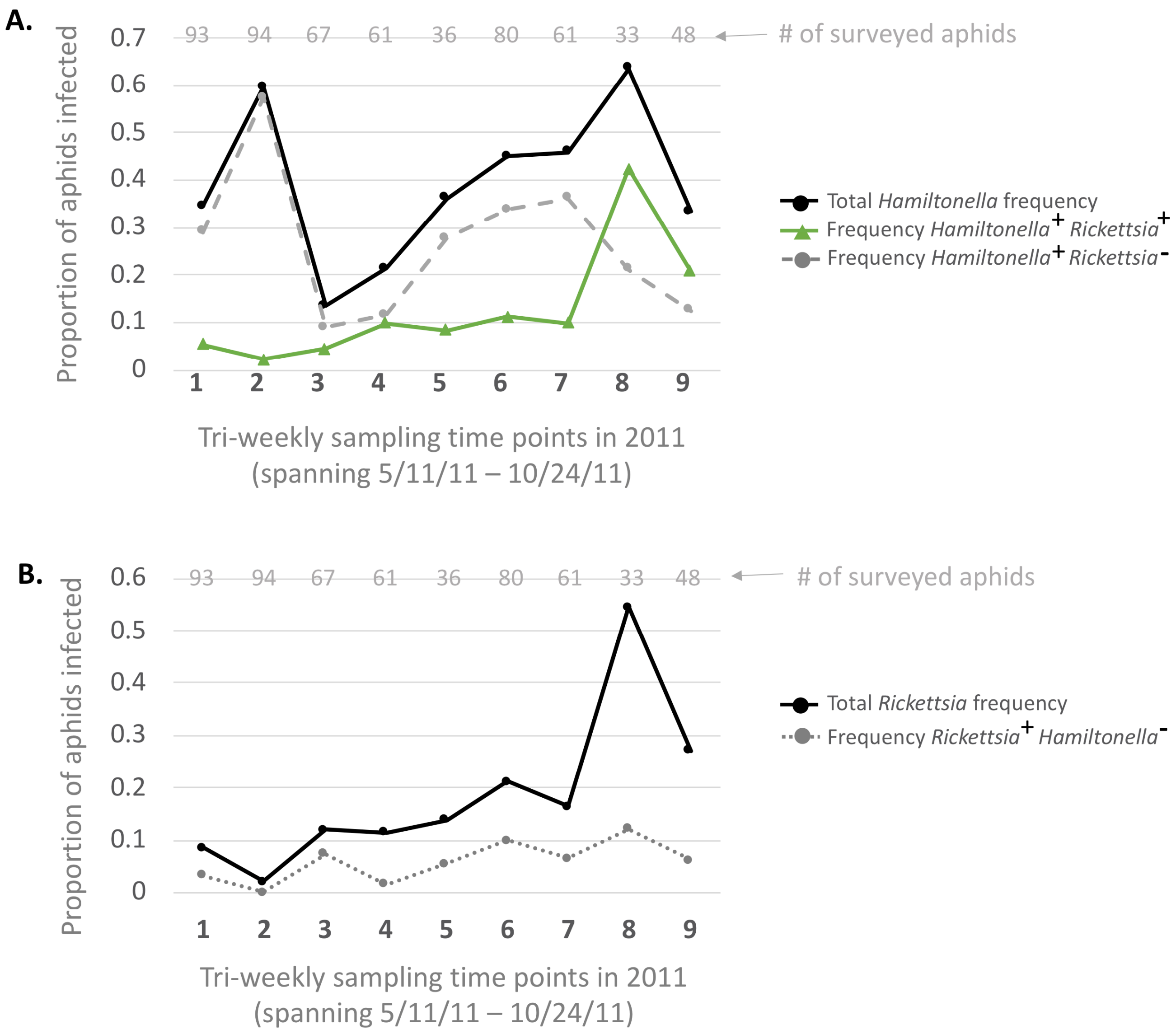


**Figure S5: Data from 2011 collections in nearby southeastern Pennsylvania alfalfa fields reveal potential selection on the *Hamiltonella-Rickettsia* co-infection in the later periods of the growing season.** Data taken from Appendix S1 of Smith et al. 2015 [64]. Only aphids screened for both *Hamiltonella* and *Rickettsia* in alfalfa fields of southeastern Pennsylvania were included. Graphing scheme follows that used for **Figures 4 & 5**. Panel A: *Hamiltonella*-focused plots. Panel B: *Rickettsia*-focused plots. Patterns across time points 7 & 8 (September 12 – October 3) show: 1) large increases in the frequencies of both symbionts (+17.7% for Ham; +38.2% for Rick); 2) strong increases in the frequencies of aphids harboring both symbionts (+32.6% for Ham+Rick+ aphids); and 3) no strong increases in frequencies of aphids harboring one symbiont without the other (-14.9% for Ham+Rick-; +5.6% for Rick-Ham+). Combined, these patterns suggest selection on the *Hamiltonella-Rickettsia* co-infection unfolded between times 7-8. By similar logic it may have also been selection against this co-infection between time points 8 & 9, although the evidence is slightly less convincing.
